# Modeling Hemolytic-Uremic Syndrome: In-Depth Characterization of Distinct Murine Models Reflecting Different Features of Human Disease

**DOI:** 10.3389/fimmu.2018.01459

**Published:** 2018-06-25

**Authors:** Sophie Dennhardt, Wiebke Pirschel, Bianka Wissuwa, Christoph Daniel, Florian Gunzer, Sandro Lindig, Anna Medyukhina, Michael Kiehntopf, Wolfram W. Rudolph, Peter F. Zipfel, Matthias Gunzer, Marc Thilo Figge, Kerstin Amann, Sina M. Coldewey

**Affiliations:** ^1^Department of Anesthesiology and Intensive Care Medicine, Jena University Hospital, Jena, Germany; ^2^Septomics Research Center, Jena University Hospital, Jena, Germany; ^3^Center for Sepsis Control and Care, Jena University Hospital, Jena, Germany; ^4^Department of Nephropathology, Friedrich-Alexander University (FAU) Erlangen-Nürnberg, Erlangen, Germany; ^5^Institute of Medical Microbiology and Hygiene/Institute of Virology, TU Dresden, Dresden, Germany; ^6^Applied Systems Biology, Leibniz Institute for Natural Product Research and Infection Biology, Hans Knöll Institute, Leibniz-Association, Jena, Germany; ^7^Department of Clinical Chemistry and Laboratory Medicine, Jena University Hospital, Jena, Germany; ^8^Department of Infection Biology, Leibniz Institute for Natural Product Research and Infection Biology, Jena, Germany; ^9^Institute for Experimental Immunology and Imaging, University Hospital, University Duisburg-Essen, Essen, Germany; ^10^Friedrich Schiller University (FSU) Jena, Jena, Germany

**Keywords:** hemolytic-uremic syndrome, Shiga toxin, enterohemorrhagic *E. coli*, *Escherichia coli*, acute kidney injury, mouse models, Shiga-toxin-producing *Escherichia coli*

## Abstract

Diarrhea-positive hemolytic-uremic syndrome (HUS) is a renal disorder that results from infections with Shiga-toxin (Stx)-producing *Escherichia coli*. The aim of this study was to establish well-defined refined murine models of HUS that can serve as preclinical tools to elucidate molecular mechanisms of disease development. C57BL/6J mice were subjected to different doses of Stx2 purified from an *E. coli* O157:H7 patient isolate. Animals received 300 ng/kg Stx2 and were sacrificed on day 3 to establish an acute model with fast disease progression. Alternatively, mice received 25 ng/kg Stx2 on days 0, 3, and 6, and were sacrificed on day 7 to establish a subacute model with moderate disease progression. Indicated by a rise in hematocrit, we observed dehydration despite volume substitution in both models, which was less pronounced in mice that underwent the 7-day regime. Compared with sham-treated animals, mice subjected to Stx2 developed profound weight loss, kidney dysfunction (elevation of plasma urea, creatinine, and neutrophil gelatinase-associated lipocalin), kidney injury (tubular injury and loss of endothelial cells), thrombotic microangiopathy (arteriolar microthrombi), and hemolysis (elevation of plasma bilirubin, lactate dehydrogenase, and free hemoglobin). The degree of complement activation (C3c deposition), immune cell invasion (macrophages and T lymphocytes), apoptosis, and proliferation were significantly increased in kidneys of mice subjected to the 7-day but not in kidneys of mice subjected to the 3-day regime. However, glomerular and kidney volume remained mainly unchanged, as assessed by 3D analysis of whole mount kidneys using CD31 staining with light sheet fluorescence microscopy. Gene expression analysis of kidneys revealed a total of only 91 overlapping genes altered in both Stx2 models. In conclusion, we have developed two refined mouse models with different disease progression, both leading to hemolysis, thrombotic microangiopathy, and acute kidney dysfunction and damage as key clinical features of human HUS. While intrarenal changes (apoptosis, proliferation, complement deposition, and immune cell invasion) mainly contribute to the pathophysiology of the subacute model, prerenal pathomechanisms (hypovolemia) play a predominant role in the acute model. Both models allow the further study of the pathomechanisms of most aspects of human HUS and the testing of distinct novel treatment strategies.

## Introduction

Hemolytic-uremic syndrome (HUS) is a form of thrombotic microangiopathy that mainly affects the kidneys. Initial renal endothelial cell damage is a common pathological feature of all HUS variants, of which the causes may differ (1). Infection-induced HUS can develop as a serious systemic complication following infections with distinct pathogens, including Shiga-toxin (Stx)-producing *Escherichia coli* (STEC) (2, 3) or pneumococcal-surface protein-C-expressing *Streptococcus pneumoniae* (4) whereas atypical or genetic HUS results from autoimmune or genetic defects, leading to altered complement regulation (5).

Hemolytic-uremic syndrome is the leading cause of acute renal failure in children (6), in which the proportion of STEC-HUS is estimated as 85–90% in comparison to atypical HUS with 5–10% and *S. pneumoniae*-HUS with approximately 5% (4, 7). The prognosis of atypical HUS has markedly improved as administration of a humanized monoclonal antibody against complement C5 allows the reversal of renal pathology and, thus, the prevention of end-stage renal disease (8). By contrast, the pathophysiological principles of infection-associated HUS are less well understood and specific biomarkers and prognosis-improving treatable targets are not available to date. However, a large outbreak of HUS caused by Stx2-producing O104:H4 *E. coli* in Germany in 2011 emphasized the impact of this pathogen on public health. A total of 3,861 cases, including 54 deaths, were reported within 2 months, of which 845 developed HUS, consuming tremendous resources in critical care units across Northern Germany (9). Prospective randomized studies investigating STEC infections and HUS are difficult to conduct in humans owing to the sporadic nature of the disease and the unpredictable occurrence of outbreaks. Therefore, there is urgent demand for animal models to study pathophysiological aspects of HUS systematically.

Bacterial Stxs, of which the two major types Stx1 and Stx2 can be discriminated, are acknowledged as key virulence factors of STEC (10). Epidemiological data revealed that Stx2 plays the predominant role in development of hemorrhagic colitis and HUS (11–14). Stxs are typical AB5 toxins. The five B subunits bind to the glycolipid receptor globotriaosylceramide (Gb3) on target cells. The toxin is then internalized (15) and the A subunit, activated by intracellular proteases, exerts N-glycosidase activity hereby deactivating mammalian ribosomes (16) and inhibiting protein synthesis leading to cell death (3). According to current understanding, a pro-thrombotic environment caused by initial endothelial cell injury and subsequent transcription events leading to generation or release of inflammatory cytokines, chemokines, and adhesion molecules, paves the way for development of thrombotic microangiopathy in the kidney with inappropriate deposition of clots in the microvasculature and subsequent tissue ischemia and organ injury [reviewed in Ref. (17, 18)]. Several related mechanisms can lead to the same endothelial injury in different forms of HUS. Thus, comparable to other syndromes associated with excessive endothelial damage, such as sepsis, the pathophysiology of HUS is complex.

Since Karmali linked sporadic cases of HUS with cytotoxin-producing *E. coli* isolated from patient feces in a breakthrough discovery in 1983 (2), numerous *in vivo* models have been developed to study this syndrome, applying such different animals as non-human primates (19, 20), gnotobiotic piglets (21–25), rabbits (26, 27), rats (28–30), and mice (31–34). Reportedly, certain aspects of HUS, such as, thrombotic microangiopathy, glomerular damage, and thrombocytopenia, are difficult to reproduce in murine models (32, 33). Nevertheless, to conduct mechanistic and early interventional studies requiring investigation of relevant signaling pathways by inactivation or overexpression of certain genes, well-defined and reproducible mouse models mimicking key characteristics of HUS are urgently needed.

Identification of novel pathomechanisms and treatable targets in different stages of STEC-HUS carries the potential for development of novel therapeutic strategies. This study was designed to establish refined murine *in vivo* models with an acute and a subacute disease progression to create better tools to further elucidate molecular mechanisms underlying HUS pathogenesis and to prospectively conduct pharmacological studies. The pathology was induced by intravenous (i.v.) application of Stx2 that was purified from the well-characterized O157:H7 patient isolate of enterohemorrhagic *E. coli* (EHEC) 86-24 (35). Here, we established and characterized in-depth two HUS models in C57BL/6J mice that differed conceptually by the endpoint of the experiment and the dose and regimen of Stx2 application. We attenuated severe hypovolemia—for the first time in murine HUS models—by regular intermittent subcutaneous (s.c.) administration of balanced electrolyte solutions, thereby mimicking supportive therapy in common clinical practice, thus increasing the clinical relevance of our models.

## Materials and Methods

### Purification and Characterization of Stx2

Stx2 was purified on different ion exchange columns and finally on a gel filtration column by our own chromatographic procedure from liquid cultures of the Stx2-producing O157:H7 EHEC strain 86-24 (35). Bacteria were grown in 5 l Luria-Bertani medium starting from an overnight culture at 1:1,000 dilution. After 4 h of incubation, mitomycin C (Sigma-Aldrich Chemie, Munich, Germany) was added to a final concentration of 0.4 mg/l to enhance the Stx release from the bacteria. Incubation was continued for another 20 h. Cultures were then centrifuged for 20 min at 16,000 *g* in an ultracentrifuge using 500 ml polycarbonate beakers in a JA-10 fixed angle rotor (Beckman Coulter, Krefeld, Germany). The supernatant was kept and sterile-filtered (Corning 1,000 ml Vacuum Filter/Storage Bottle System, 0.22 µm Pore; Sigma-Aldrich Chemie). A 70% ammonium sulfate precipitation was carried out at 4°C and the precipitate isolated by centrifugation. The precipitate was dissolved in a low salt buffer at pH 6.2 and dialyzed against 5 mM phosphate/NaCl buffer for 48 h at 4°C (Slide-A-Lyzer Dialysis Cassettes, 10,000 MWCO, 30 ml; Thermo Fisher Scientific, Dreieich, Germany). The crude protein solution was applied on an anion exchange column (HiTrap Q HP XL; GE Healthcare Europe, Freiburg, Germany) using an ÄKTA pure 25 l chromatography system (GE Healthcare Europe) and the protein was eluted with 1 M NaCl buffer at pH 6.2. The appropriate protein fractions were pooled and dialyzed against 5 mM acetate buffer at pH 3.9. The re-buffered protein solution was then applied to a cation exchange column (HiTrap SP HP XL, GE Healthcare Europe). Bound protein was eluted with 1 M NaCl buffer at pH 3.9. Again, the pooled protein fractions were dialyzed in 50 mM Tris/HCl buffer at pH 6.5. In a third chromatographic step, the purified protein solution was applied on a HiLoad 26/600 Superdex 200 prep grade column (GE Healthcare Europe) and eluted with a flow rate of 1 ml/min isocratically. The appropriate fractions were pooled again. To concentrate the toxin, pooled samples were centrifuged using Vivaspin 20 ultrafiltration devices (10,000 MWCO; Sartorius, Göttingen, Germany). Protein concentration was determined by the bicinchoninic acid method following the protocol supplied by the manufacturer (Sigma-Aldrich Chemie). This purified and concentrated Stx2 protein solution of 0.35 mg/ml was then characterized applying SDS polyacrylamide gel electrophoresis and immunoblot analysis, as described previously (36). Two subunits were detected at 32 and 7 kDa and the protein was free of any visible contamination.

### Cytotoxicity Testing of Stx2

Cytotoxicity and LD_50_ of purified Stx2 were determined using Vero cells stained with the vital dye neutral red. 1 × 10^4^ Vero cells per well in 200 µl DMEM/10% FBS (Thermo Fisher Scientific) were seeded into a 96-well plate and grown at 37°C/5% CO_2_ for 1 day. 10 µl cell culture medium was replaced by 10 µl medium in duplicate, containing 1 ng to 0.01 fg Stx2 in 10-fold dilutions. Cells were incubated further at 37°C/5% CO_2_ for 2 days. Then, vital staining was performed with the “*In vitro* Toxicology Assay Kit Neutral Red based” (Sigma-Aldrich Chemie) according to the manufacturer’s instructions. Dye release was assessed spectrophotometrically (Tecan Sunrise™ 96-well Absorbance Microplate Reader; Tecan Deutschland, Crailshaim, Germany) by measuring absorbance at a wavelength of 540 nm against the background absorbance of multiwell plates, measured at 690 nm. Values obtained from wells containing only cell culture medium were set at LD_100_, values from Vero cell monolayers without Stx2 treatment were set at LD_0_, and the LD_50_ was calculated from it.

### Dose-Response Study for Stx2 *In Vivo*

Male C57BL/6J mice (aged 10–16 weeks, 20–30 g BW) were intravenously subjected to a single dose of either 50, 100, 150, or 300 ng/kg BW Stx2 in 5 ml/kg NaCl 0.9%, subsequently weighed every 24 h and scored every 12 h to monitor disease progression. After 72 h, mice were exsanguinated in deep ketamine (100 mg/kg BW in 5 ml/kg NaCl 0.9% i.p.) and xylazine (10 mg/kg BW in 5 ml/kg NaCl 0.9% i.p.) anesthesia. Blood was obtained by puncturing the *v. cava* to compile hemograms and analyze kidney dysfunction by measuring plasma urea and creatinine.

### Animal Experiments

Male wild-type C57BL/6J mice (aged 10–16 weeks, 20–30 g BW) were randomly assigned to one of the two treatment groups (sham, Stx2). Mice were kept under standardized laboratory conditions, and received standard rodent chow and water *ad libitum*. HUS was induced by either a single i.v. injection of Stx2 (300 ng/kg BW in 5 ml/kg NaCl 0.9%; acute model) or three i.v. injections of Stx2 (25 ng/kg BW in 5 ml/kg BW NaCl 0.9% each; subacute model) on days 0, 3, and 6. Sham animals received 5 ml/kg BW NaCl 0.9% only. Mice were weighed every 24 h and scored every 12 h (acute model) or 8 h (subacute model) to monitor disease progression. To prevent the severe dehydration observed in the dose-response study, mice received 500 µl of Ringer’s lactate s.c. every 12 h (acute model) or 800 µl Ringer’s lactate s.c. every 8 h (subacute model). 72 h (acute model) or 168 h (subacute model) after HUS induction, mice were sacrificed by exsanguination in deep anesthesia (see Dose-Response Study for Stx2 *In Vivo*).

### Evaluation of Disease Progression

Disease progression was monitored by both weight loss and scoring. A scoring system based on the activity of mice was used (ranging from 1—normally active, 2—active with slight restrictions, 3—active with clear intermissions, 4—slowed, 5—lethargic, 6—moribund, to 7—dead). In the course of the experiments, a modified HUS score that additionally included neurological symptoms was established and tested in the subacute model (Table S1 in Supplementary Material).

### Blood Analysis

Lithium heparin anti-coagulated blood was obtained and hemograms were compiled using the pocH100iV system (Sysmex, Kobe, Japan). According to the manual Sysmex pocH100iV generally distinguishes between small (W-SCC = small cell count, lymphocytes), middle (W-MCC = middle cell count; monocytes, basophils, eosinophils), and large (W-LCC = large cell count; neutrophils) leukocytes. However, our setup for mice only determines small and large cell count; middle-sized cells are included in the small cell count. Afterward, blood was centrifuged at 3,000 *g* for 15 min at room temperature to obtain plasma. Alanine aminotransferase (ALAT), aspartate aminotransferase (ASAT), bilirubin, lactate dehydrogenase (LDH), urea, and creatinine were measured using an Architect c16200/ci8200 automated clinical chemistry system (Abbott Diagnostics, Abbott Park, IL, USA) according to the manufacturer’s recommendations. Plasma neutrophil gelatinase-associated lipocalin (NGAL) was measured using an ELISA kit provided by BioLegend (San Diego, CA, USA) according to the manufacturer’s protocol (sham samples were diluted 1:200 and Stx2 samples 1:1,000 due to the assay range).

Hemolysis was quantified (scoring: 0: no hemolysis, absorbance = 0; 1: up to 5% hemolysis, absorbance <0.04; 2: >5–20% hemolysis, absorbance <0.12; 3: >20–50% hemolysis, absorbance <0.16; 4: >50% hemolysis, absorbance >0.16) by measuring hemoglobin absorption spectra (at 500–600 nm) in plasma with a Spark^®^ multimode reader (Tecan, Maennedorf, Swiss) and comparing the intensity to a standard.

### Tissue Preparation

At the end of the experiment—after puncture of the *v. cava*—anesthetized mice were perfused with 0.9% NaCl *via* the left ventricle and right atrium for removal of erythrocytes within the tissue. Afterward, kidneys were removed and fixed instantly with 5% buffered formaldehyde solution for at least 72 h at 4°C, processed, and subsequently embedded in paraffin blocks.

### Histopathology

Renal sections (1 µm) were deparaffinized and subjected to routine staining: hematoxylin/eosin, periodic acid Schiff (PAS), and acid fuchsin orange G (SFOG). Histomorphological changes were determined on PAS stained kidney sections. Therefore, 10 cortical fields per kidney adjacent to one another were randomly graded mainly for signs of tubular injury (i.e., brush border loss, epithelial cell flattening, or vacuolization) at a magnification of 400× using a scoring system from 0 to 3: 0: no damage, 1: <25% damaged, 2: 25–50% damaged, 3: >50% damaged. Two trained investigators independently evaluated the sections without knowledge of the experimental group.

### Immunohistochemical Staining

Renal sections were deparaffinized and hydrated in a descending series of ethanol (3× 5 min xylene, 2× 1 min 100% ethanol, 2× 1 min 96% ethanol, 1× 1 min 70% ethanol, aqua dest.). Endogenous peroxidase activity was blocked by incubating sections in 3% H_2_O_2_ for 15 min at room temperature. Antigen retrieval was performed in target retrieval solution (pH 6; Dako, Glostrup, Denmark) for 2.5 min at 120°C using a pressure cooker. Nonspecific binding sites were subsequently saturated in serum or blocked with avidin/biotin solution. All tissue sections were incubated with a primary antibody overnight at 4°C in appropriate dilutions (Table S2 in Supplementary Material). Sections were further processed using the VectaStain ABC kit (Vector Laboratories, Burlingame, CA, USA) according to the manufacturer’s recommendations and 3,3-diaminobenzidine (DAB) as substrate. Finally, the sections were counterstained in hemalaun, dehydrated, and mounted for observation. Immunohistochemistry images were acquired using an Olympus Bx60 microscope equipped with an XC30 camera at various magnifications. Images were taken after white balance, auto exposure, and introduction of scale bar using cellSens software (Olympus Deutschland, Hamburg, Germany). No further adjustments or signal amplifications were performed.

### Quantification of Immunodetection

A grid superimposed on 20 (for F4-80, CD3, cleaved caspase 3 and Ki67) or 30 (for CD31) cortical areas (adjacent to one another) of each section was used for quantification. While for F4-80 and Ki67 the number of intersections overlapping the positive brown staining was counted in each grid, for CD31 the number of positive caskets per grid was counted. For CD3 and cleaved caspase 3, the numbers of positive cells per mm^2^ was calculated based on the number of positive cells counted per grid area (grid area = 0.0625 mm^2^). To analyze the expression of kidney injury molecule-1 (KIM-1) and C3c, a scoring system from 0 to 3 (0: <25%, 1: 25–50%, 2: 50–75%, 3: >75% strong positive staining per visual field) was used to evaluate 12 visual fields per section in a blinded manner (magnification: 20×).

### Ultrastructural Analysis

Ultrastructural analysis using electron microscopy was performed as described previously (37).

### Microarray Preparation and Analysis

Total kidney RNA was extracted from approximately 30 mg of kidney tissue using an RNeasy Mini kit (QIAGEN, Santa Clarita, CA, USA) according to the manufacturer’s instructions. 200 ng of total RNA was processed with the GeneChip™ Hybridization, Wash, and Stain Kit (Affymetrix, Santa Clara, CA, USA) according to the manufacturer’s protocol and hybridized to GeneChip™ mouse 2.0 ST arrays (Affymetrix) that were subsequently scanned with the GeneChip™ Scanner 3000 7G (Affymetrix). CEL files were pre-processed with Robust Multi-Array Average algorithm and background correction using R statistics software and Bioconductor packages. Intra- vs. interclass differences were assessed *via* one-way ANOVA. Genes with absolute log2-fold change >1 and *p*-values <0.05 were considered significantly differentially expressed. False discovery rate-based *p*-value adjustment was performed *via* the Benjamini–Hochberg method. A list of candidate genes was generated by identifying the overlapping differentially expressed genes (DEGs) of both models. This list was further analyzed using the Database for Annotation, Visualization, and Integrated Discovery 6.8 [DAVID 6.8 (38)] with background set to *Mus musculus* and classification stringency set to medium.

### Statistics

All values are depicted as mean ± SD of *n* observations (*n* representing number of animals studied) unless stated otherwise. Statistical analysis was performed using GraphPad 7.03 (GraphPad Software, San Diego, CA, USA). Outliers were removed using the ROUT test (*Q* = 5%). Statistics were performed as *t*-test for parametric data and Mann–Whitney *U*-test for non-parametric data. A *p*-value <0.05 was considered significant.

## Results

### Cytotoxicity of Stx2

Cytotoxicity of Stx2 purified from EHEC 86-24 was measured *in vitro* in Vero cells by neutral red assay. The LD_50_ in this cell line is 9.51 pg/ml (Figure S1 in Supplementary Material).

### Dose-Response Relationship of Stx2 *In Vivo*

**Figure 1:**
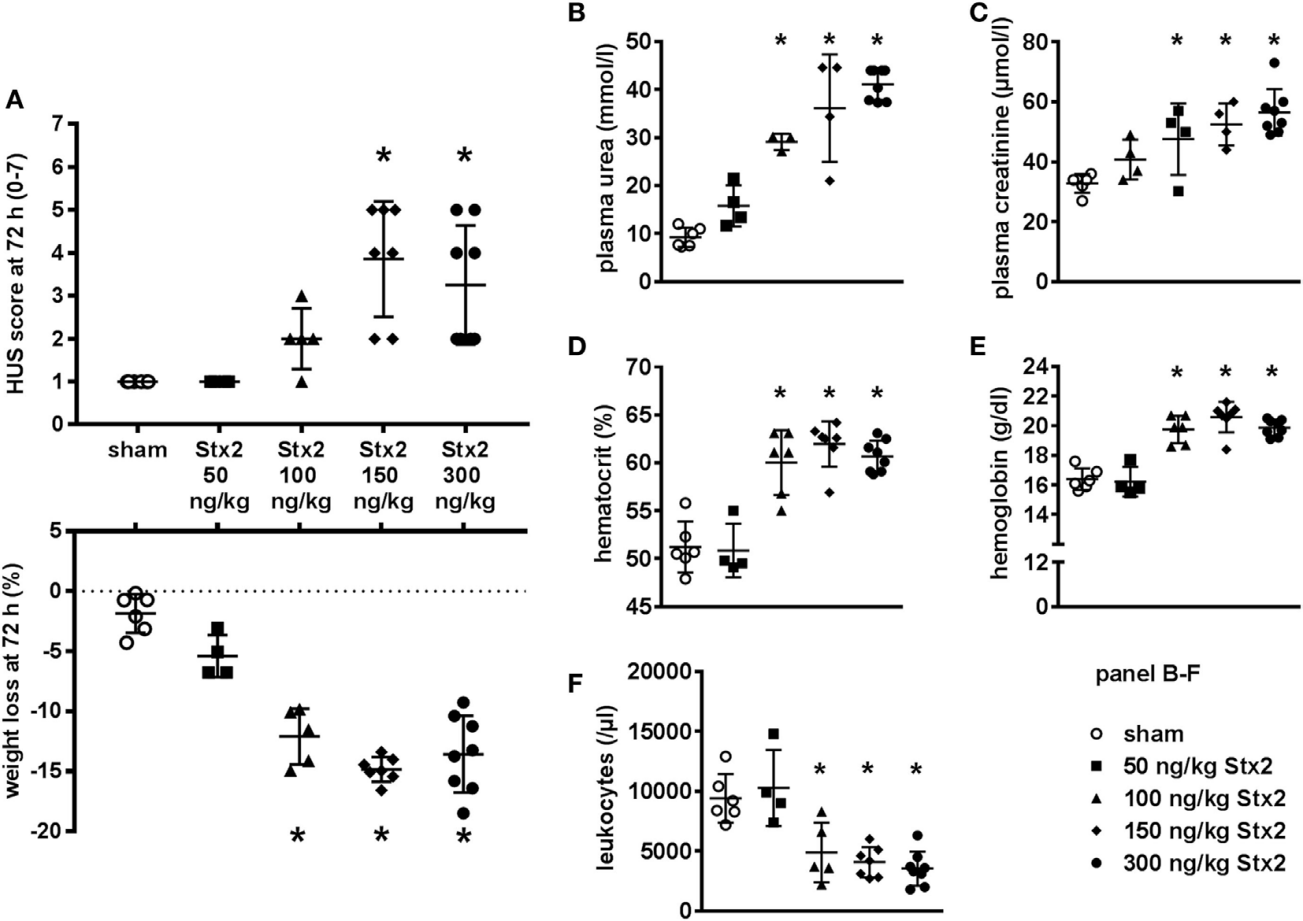
Dose-response study for Stx2 in C57BL/6J mice. **(A–F)** Mice were subjected to different Stx2 concentrations. The effect was assessed after 72 h. Data are expressed as mean ± SD for *n* observations, *p < 0.05 vs. sham. **(A)** Final score and overall weight loss at 72 h (score: Kruskal–Wallis test with Dunn’s correction, weight loss: one-way ANOVA with Bonferroni *post hoc* test; sham *n* = 6, 50 ng/kg BW Stx2 *n* = 4, 100 ng/kg BW Stx2 *n* = 5, 150 ng/kg BW Stx2 *n* = 7, 300 ng/kg BW Stx2 *n* = 8). Kidney function was evaluated by **(B)** plasma urea and **(C)** plasma creatinine. **(D)** Hematocrit and **(E)** hemoglobin were measured as indicators of red blood cell count. Immune response was monitored by **(F)** white blood cell count. **(B–F)** Groups: sham *n* = 6, 50 ng/kg Stx2 *n* = 4, 100 ng/kg Stx2 *n* = 6, 150 ng/kg Stx2 *n* = 7, 300 ng/kg Stx2 *n* = 8 (one-way ANOVA with Bonferroni *post hoc* test).

All parameters observed in the dose-response study followed a clear dose-dependency (Figure 1). Clinical signs of HUS reflected by significant rises in HUS score and weight loss were detectable in mice challenged with Stx2 doses higher than 100 ng/kg BW (HUS score) and 50 ng/kg BW (weight loss), respectively (Figure 1A). Plasma urea and creatinine as markers of kidney dysfunction (Figures 1B,C) also showed dose-dependency with only slight elevations in mice subjected to 50 ng/kg BW Stx2 and highest levels in mice challenged with 300 ng/kg BW. Hematocrit and hemoglobin (Figures 1D,E) as indicators of hemoconcentration dose-dependently increased in mice challenged with more than 50 ng/kg BW Stx2. White blood cell count (Figure 1F) was decreased in animals challenged with higher doses of Stx2, indicating leukopenia. According to these results, a single dose of 300 ng/kg BW Stx2 was chosen to establish the acute model as it reproducibly induced kidney dysfunction. To develop a subacute model, an application regime of 3× 25 ng/kg BW Stx2 was chosen, as a single dose was not expected to cause significant or lethal symptoms in mice and the accumulated total dose of 75 ng/kg BW Stx2 should be able to induce moderate signs of disease.

### Effect of Different Stx2 Regimens on Clinical Presentation

**Figure 2:**
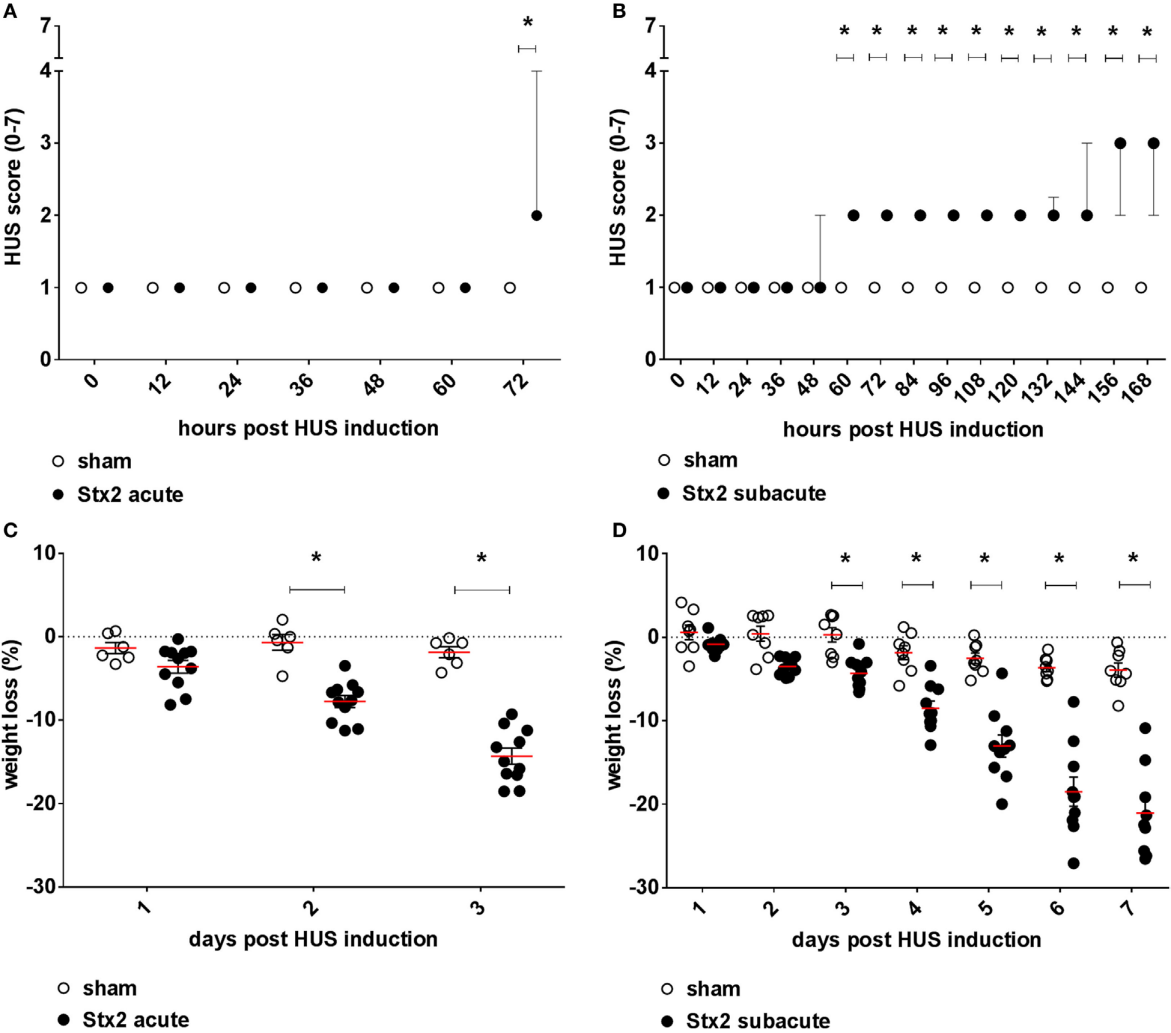
Clinical presentation of C57BL/6J mice subjected to different Stx2 regimens. Clinical presentation was assessed by a score ranging from 1 = very active to 7 = dead for the **(A)** acute model (sham *n* = 6, Stx2 *n* = 11) and **(B)** subacute model (sham *n* = 8, Stx2 *n* = 10). **(A,B)** Data are expressed as median ± interquartile range for *n* observations. *p < 0.05 for sham vs. Stx2 at each respective time point (Mann–Whitney *U*-test). Weight loss of C57BL/6J wild-type mice was assessed every 24 h for the **(C)** acute model (sham *n* = 6, Stx2 *n* = 11) and the **(D)** subacute model (sham *n* = 8, Stx2 *n* = 10). **(C,D)** Data are expressed as mean ± SD for *n* observations. *p < 0.05 for sham vs. Stx2 at each respective time point (two-way ANOVA with Bonferroni *post hoc* test).

Mice challenged with 300 ng/kg Stx2 did not exhibit a decrease in activity until just before the endpoint 72 h after HUS induction (Figure 2A). However, progression of the disease was observable by profound weight loss of mice challenged with Stx2 reaching significance from day 2 after initial Stx2 injection when compared with sham-treated animals (Figure 2C). At day 3, mice lost approximately 20% of their body weight and exhibited tremors and ataxia. By contrast, mice challenged with 3× 25 ng/kg of Stx2 were significantly less active starting from 60 h after HUS induction onward compared with the sham group (Figure 2B). Of note, one of the animals challenged with 3× 25 ng/kg Stx2 was already moribund 144 h after HUS induction and therefore euthanized and excluded from further analysis. Significant profound weight loss was also observed in mice challenged with 3× 25 ng/kg of Stx2 compared with sham animals from day 3 after initial Stx2 injection (Figure 2D). At the endpoint of the subacute model, critically ill mice exhibited tremor, ataxia, and a pronounced hind limb clasping reflex as a sign of neurological involvement. We attempted to further refine our activity-based score in the course of the experiment by including neurological symptoms, weight loss, and fur quality, and tested the modified score in the subacute model (Figure S2 in Supplementary Material).

### Effect of Different Stx2 Regimens on Hemoconcentration and Thrombocytes

**Figure 3:**
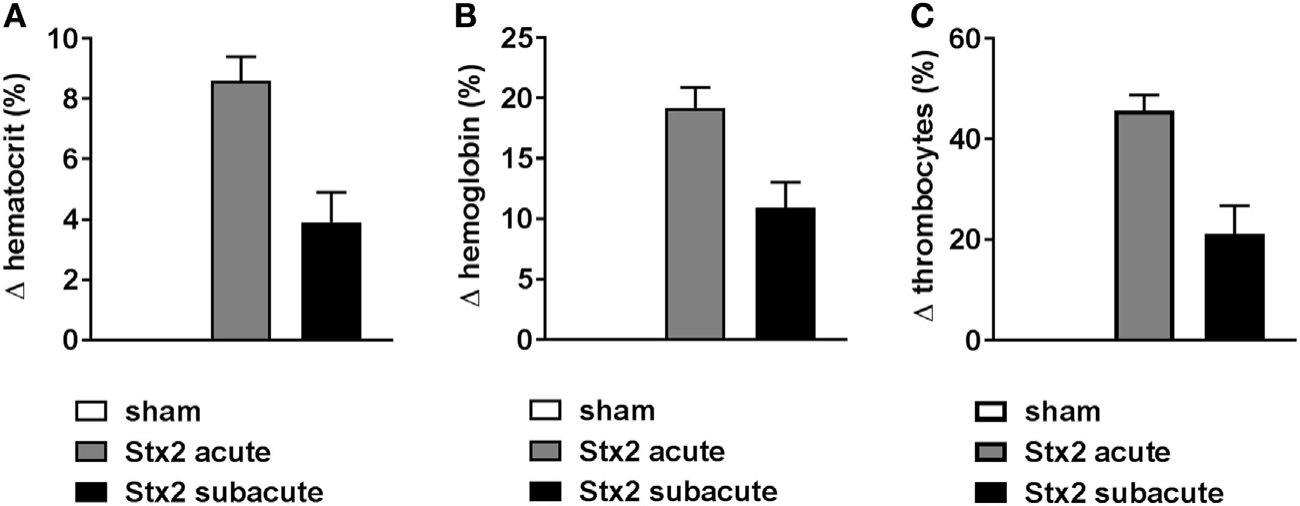
Hemoconcentration and thrombocytes in C57BL/6J mice subjected to different Stx2 regimens. Percentage change of **(A)** hematocrit, **(B)** hemoglobin, and **(C)** thrombocytes for the acute (sham *n* = 6, Stx2 *n* = 11) and subacute model (sham *n* = 5, Stx2 *n* = 6) compared with the respective sham group. **(A–C)** Data are expressed as mean ± SEM for *n* observations.

Hemoconcentration occurred in Stx2-challenged mice of both models as indicated by changes in hematocrit as well as hemoglobin. However, these changes were twice as pronounced in the acute model despite the shorter duration of the model (Figure 3). At the endpoint of the acute model, the hematocrit significantly increased by 8.6% ± 2.7% (*p* < 0.0001) compared with sham controls. The increase of hematocrit was half as pronounced in mice challenged with 3× 25 ng/kg Stx2 (3.9% ± 2.5%; *p* = 0.0135; Figure 3A). Consequently, we observed significantly increased blood hemoglobin levels in the acute (19.2% ± 5.9%; *p* < 0.0001) and subacute model (10.9% ± 5.2%; *p* = 0.0023) compared with sham-treated mice (Figure 3B). In line with the hemoconcentration, a rise in the thrombocyte count was observed in Stx2-challenged mice that was again much more pronounced in the acute model (45.6% ± 3.1%; *p* = 0.0004) compared with the subacute model (21.1% ± 5.6%; *p* = 0.0131; Figure 3C). Thus, we cannot show thrombocytopenia in our models. However, slight occurrence of thrombocytopenia might be masked by hypovolemia and consecutive hemoconcentration.

### Effect of Different Stx2 Regimens on Hemolysis

**Figure 4:**
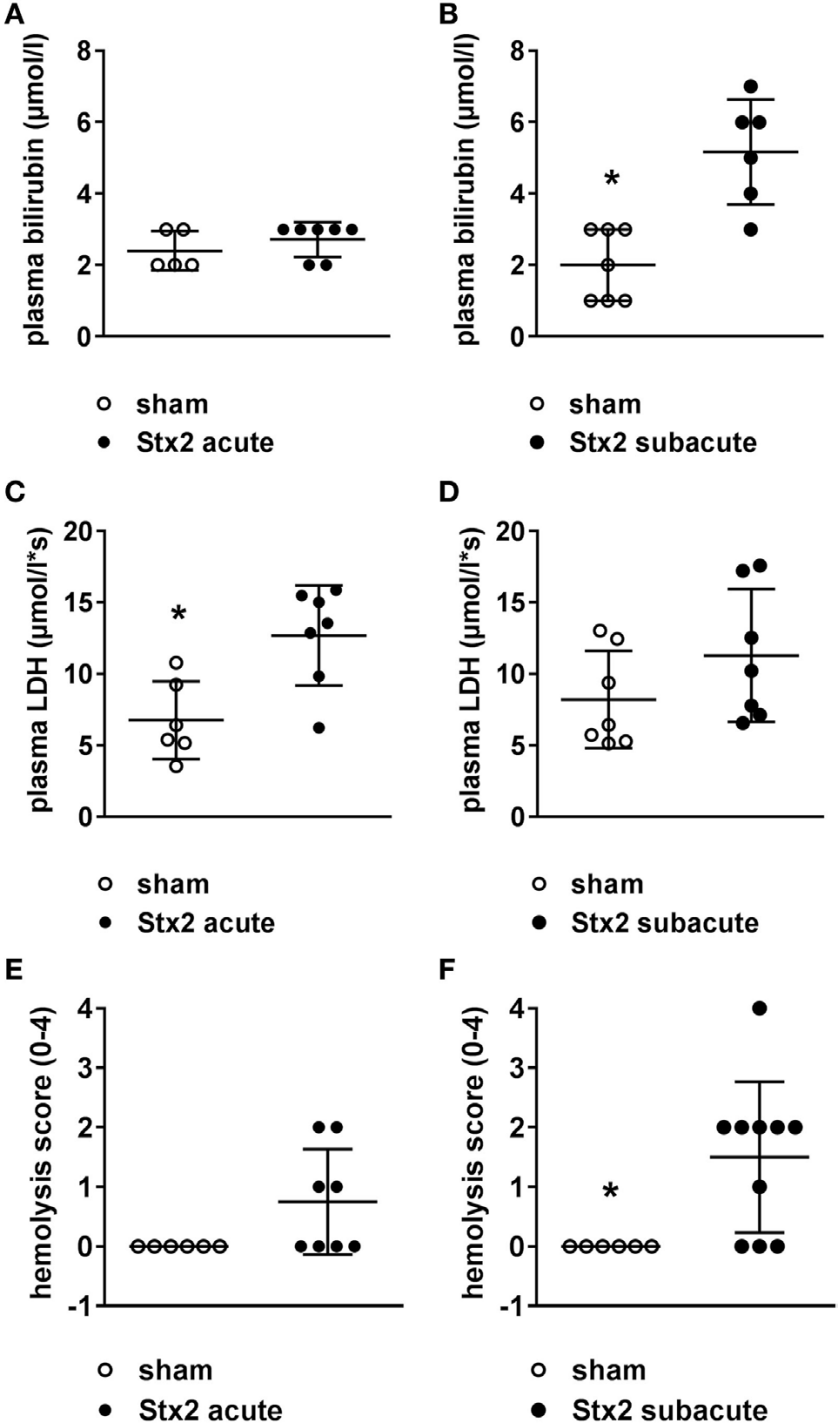
Indicators of hemolysis in C57BL/6J mice subjected to different Stx2 regimens. Plasma bilirubin levels were measured for the **(A)** acute model (sham *n* = 5, Stx2 *n* = 7) and **(B)** subacute model (sham *n* = 7, Stx2 *n* = 6). Plasma lactate dehydrogenase (LDH) levels were measured for the **(C)** acute model (sham *n* = 6, Stx2 *n* = 7) and **(D)** subacute model (sham *n* = 7, Stx2 *n* = 7). **(A–D)** Data are expressed as mean ± SD for *n* observations. *p < 0.05 sham vs. Stx2 (*t*-test). Hemolysis was photometrically quantified in plasma samples of the **(E)** acute model (sham *n* = 6, Stx2 *n* = 8) and **(F)** subacute model (sham *n* = 6, Stx2 *n* = 10). **(E,F)** Data are expressed as mean ± SD for *n* observations. *p < 0.05 for sham vs. Stx2 (Mann–Whitney *U*-test).

Both plasma LDH and bilirubin were measured as indirect hemolysis markers. While plasma LDH was significantly higher (*p* = 0.0058) in the Stx2 group of the acute model (Figures 4C,D), plasma bilirubin was significantly increased (*p* = 0.0018) only in the Stx2 group of the subacute model compared with the respective sham group (Figures 4A,B). Photometric determination of free plasma hemoglobin revealed hemolysis in Stx2-challenged animals of both models, however, to a significant extent only in the subacute model (*p* = 0.017) (Figures 4E,F).

### Effect of Different Stx2 Regimens on Immune Response

**Figure 5:**
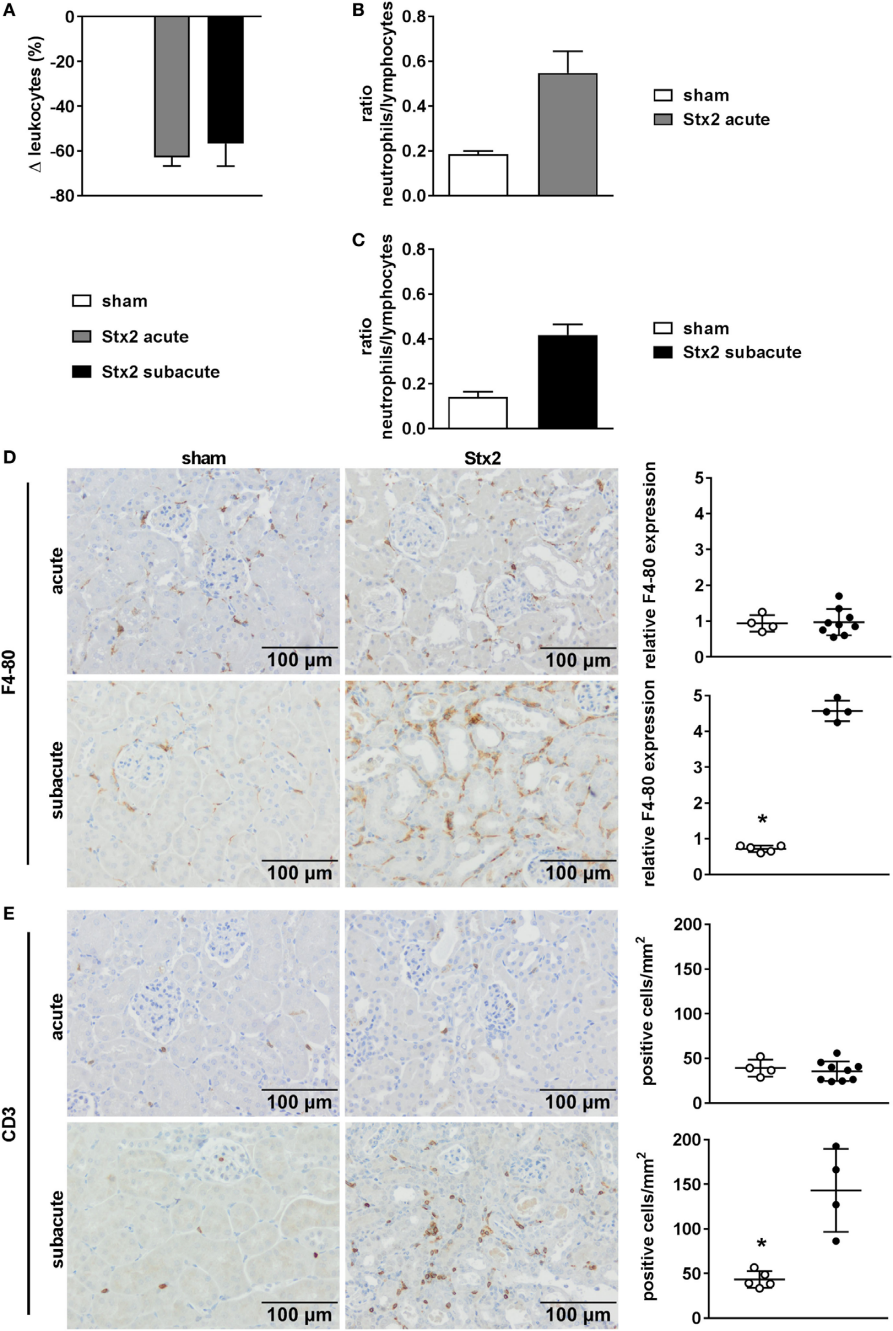
Immune response in C57BL/6J mice subjected to different Stx2 regimens. **(A)** Percentage change of leukocyte counts and **(B,C)** ratio of neutrophils to lymphocytes for the acute and subacute model compared with the respective sham group. **(A–C)** Data are expressed as mean ± SEM for *n* observations (acute: sham *n* = 6, Stx2 *n* = 11; subacute: sham *n* = 5, Stx2 *n* = 6). Representative images (scale bar 100 µm) of immunohistochemical detection and quantitative data of **(D)** F4-80 (surface antigen of macrophages) and **(E)** CD3 (surface antigen of T cells) in renal sections of C57BL/6J wild-type mice are depicted (acute: sham *n* = 4, Stx2 *n* = 9; subacute: sham *n* = 5, Stx2 *n* = 4). *p < 0.05 for sham (white dots) vs. Stx2 (black dots; *t*-test).

A significant drop in leukocyte count was found in Stx2-challenged mice in both the acute (*p* = 0.0003) and the subacute (*p* = 0.0069) model compared with the respective sham group (Figure 5A). This general leukopenia (−62.7% ± 13.0% and −56.7% ± 24.6%) was accompanied by a shift in the ratio of neutrophils to lymphocytes (including medium-sized white blood cells; acute model: 0.19 ± 0.04 in sham group vs. 0.55 ± 0.32 in Stx2 group; subacute model: 0.14 ± 0.02 in sham group vs. 0.42 ± 0.04 in Stx2 group) indicating neutrophilia and lymphocytopenia (Figures 5B,C). Immune cell invasion in the kidney was investigated by staining renal sections for F4-80 (surface marker for macrophages) and CD3 (surface marker for T lymphocytes) using immunohistochemistry. Subsequent quantification revealed no renal increase of both macrophages and T lymphocytes in the acute HUS model (Figures 5D,E, upper panels). By contrast, in the subacute model on day 7 renal infiltrations with F4-80-positive cells (*p* = 0.0079) as well as CD3-positive cells (*p* = 0.0159) were significantly increased (Figures 5D,E, lower panels).

### Effect of Different Stx2 Regimens on Kidney Dysfunction and Injury

**Figure 6:**
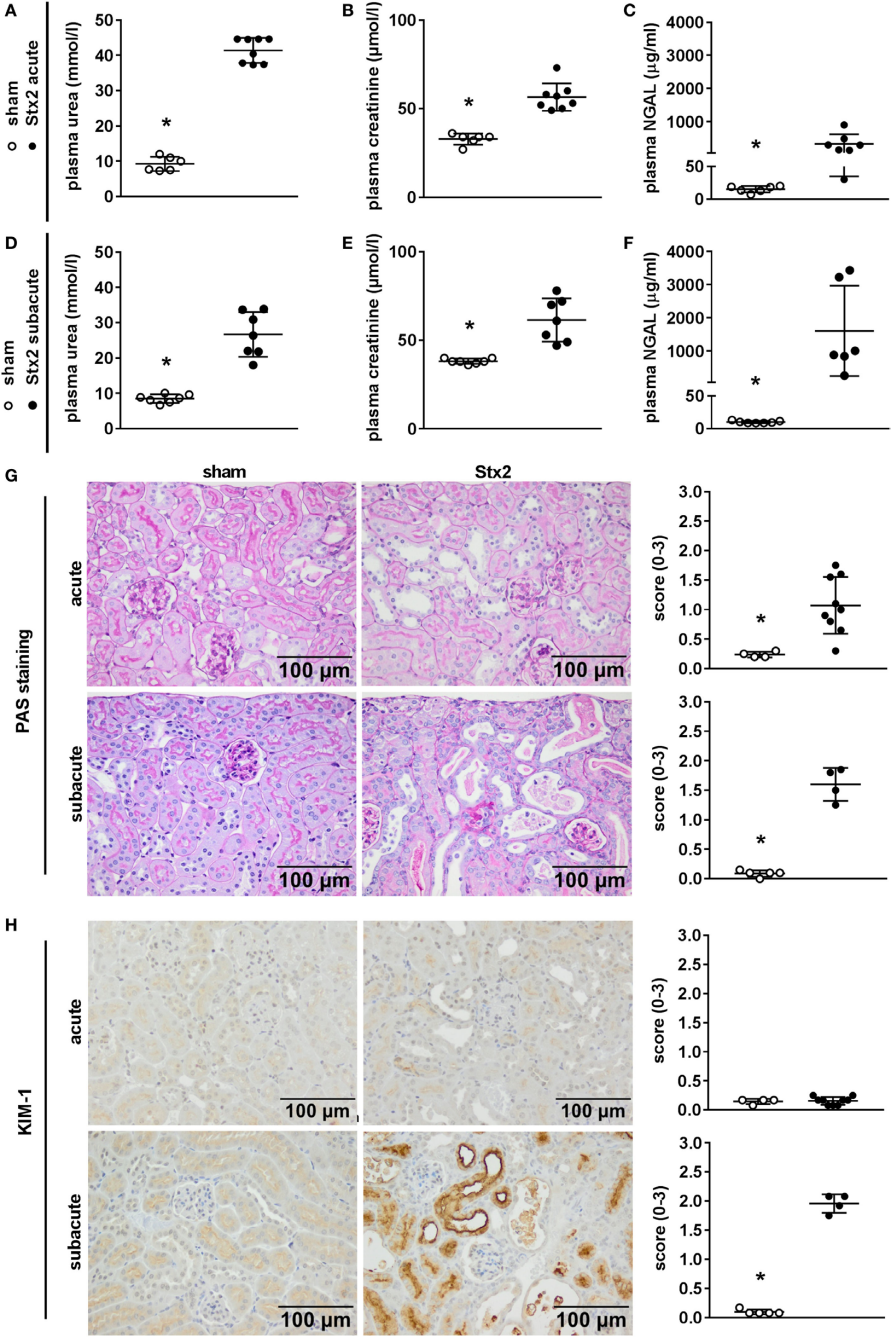
Indicators of kidney injury in mice subjected to different Stx2 regimens. **(A,D)** Plasma urea, **(B,E)** plasma creatinine, and **(C,F)** plasma neutrophil gelatinase-associated lipocalin (NGAL) levels were measured for the acute model (sham *n* = 6, Stx2 *n* = 8) and subacute model (sham *n* = 7, Stx2 *n* = 7, NGAL Stx2 *n* = 6). Data are expressed as mean ± SD for *n* observations. *p < 0.05 for sham vs. Stx2 (*t*-test). Representative images (scale bar 100 µm) and quantitative data of **(G)** periodic acid Schiff (PAS) staining and **(H)** immunohistochemical detection of kidney injury molecule-1 (KIM-1) in renal sections of C57BL/6J wild-type mice are depicted (acute: sham *n* = 4, Stx2 *n* = 9; subacute: sham *n* = 5, Stx2 *n* = 4). *p < 0.05 for sham (white dots) vs. Stx2 (black dots; Mann–Whitney *U*-test).

**Figure 7:**
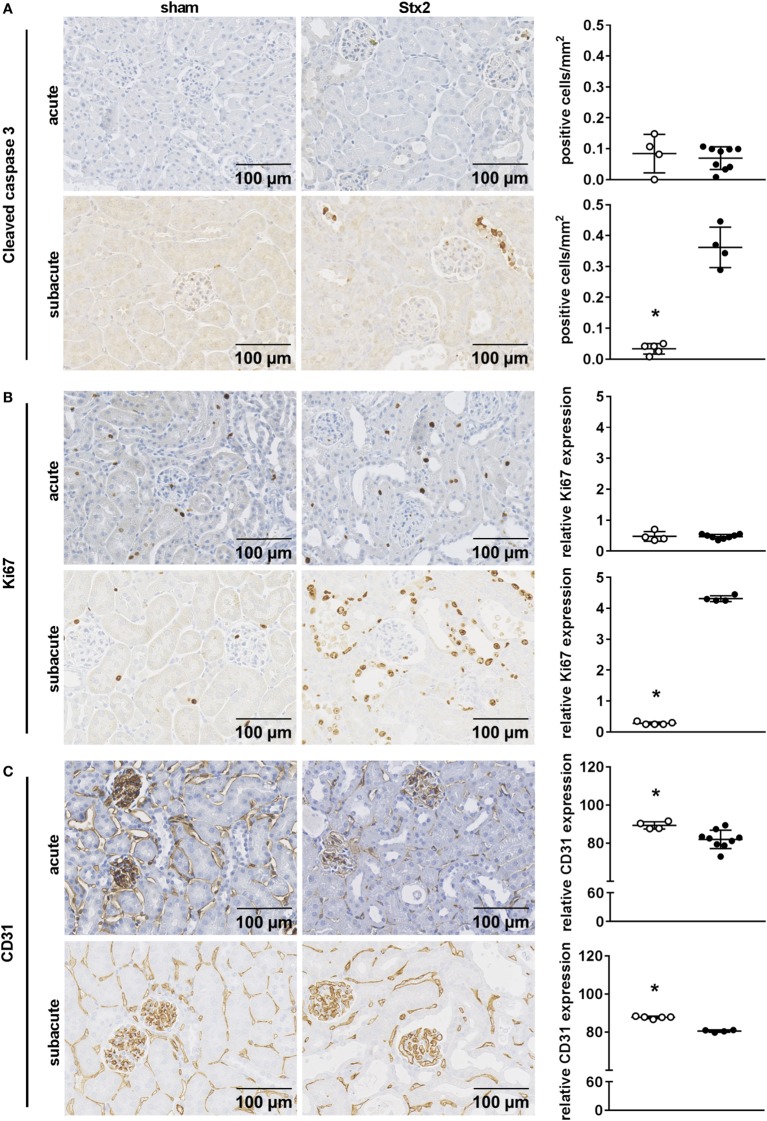
Indicators of cell death, reactive proliferation, and endothelial damage in kidney tissue of C57BL/6J mice subjected to different Stx2 regimens. Representative images of immunohistochemical detection and quantitative data of **(A)** cleaved caspase 3 (acute: sham *n* = 4, Stx2 *n* = 9; subacute: sham *n* = 5, Stx2 *n* = 3), **(B)** Ki67, and **(C)** CD31 (acute: sham *n* = 4, Stx2 *n* = 9; subacute: sham *n* = 5, Stx2 *n* = 4) in renal sections of C57BL/6J wild-type mice are depicted. *p < 0.05 for sham (white dots) vs. Stx2 (black dots; *t*-test).

We assessed kidney dysfunction and injury by laboratory markers, histomorphological, and immunohistochemical analysis. In the acute and subacute HUS model, we observed severe kidney dysfunction, indicated by a significant rise in plasma urea (Figures 6A,D), creatinine (Figures 6B,E), and NGAL (Figures 6C,F). As expected, we found no signs of liver injury evaluated by plasma ASAT and ALAT measurement (Section “Supplementary Results” and Figure S3 in Supplementary Material). Examinations of PAS stained renal sections revealed significant histomorphological changes in the acute HUS model as indicated by tubule dilatation and atrophy and occasional loss of the tubular brush border in proximal tubules (Figure 6G, upper panels). In the subacute model, these changes were more pronounced: tubular protein casts and tubular necrosis could be additionally detected (Figure 6G, lower panels). Interestingly, expression of KIM-1 was lacking in the acute HUS model but significantly increased in the subacute HUS model, markedly showing KIM-1 expression in injured tubular cells (Figure 6H). Concurrently, renal apoptosis of tubular epithelial cells, as monitored by staining for cleaved caspase 3, was only significantly increased in the subacute but not in the acute HUS model (Figure 7A). An increase in renal tubular repair, as reflected by an increased proliferative activity, was similarly exclusively observed in the Stx2 group of the subacute model (*p* = 0.0159), as assessed by evaluation of the proliferation marker Ki67 (Figure 7B). However, significantly reduced numbers of renal endothelial cells could be demonstrated by CD31 immunostaining in the acute (*p* = 0.0112) and subacute HUS model (*p* = 0.0159) (Figure 7C), indicating loss of endothelial cells by Stx2 challenge.

### Effect of Different Stx2 Regimens on Thrombus Formation and Complement Activation

**Figure 8:**
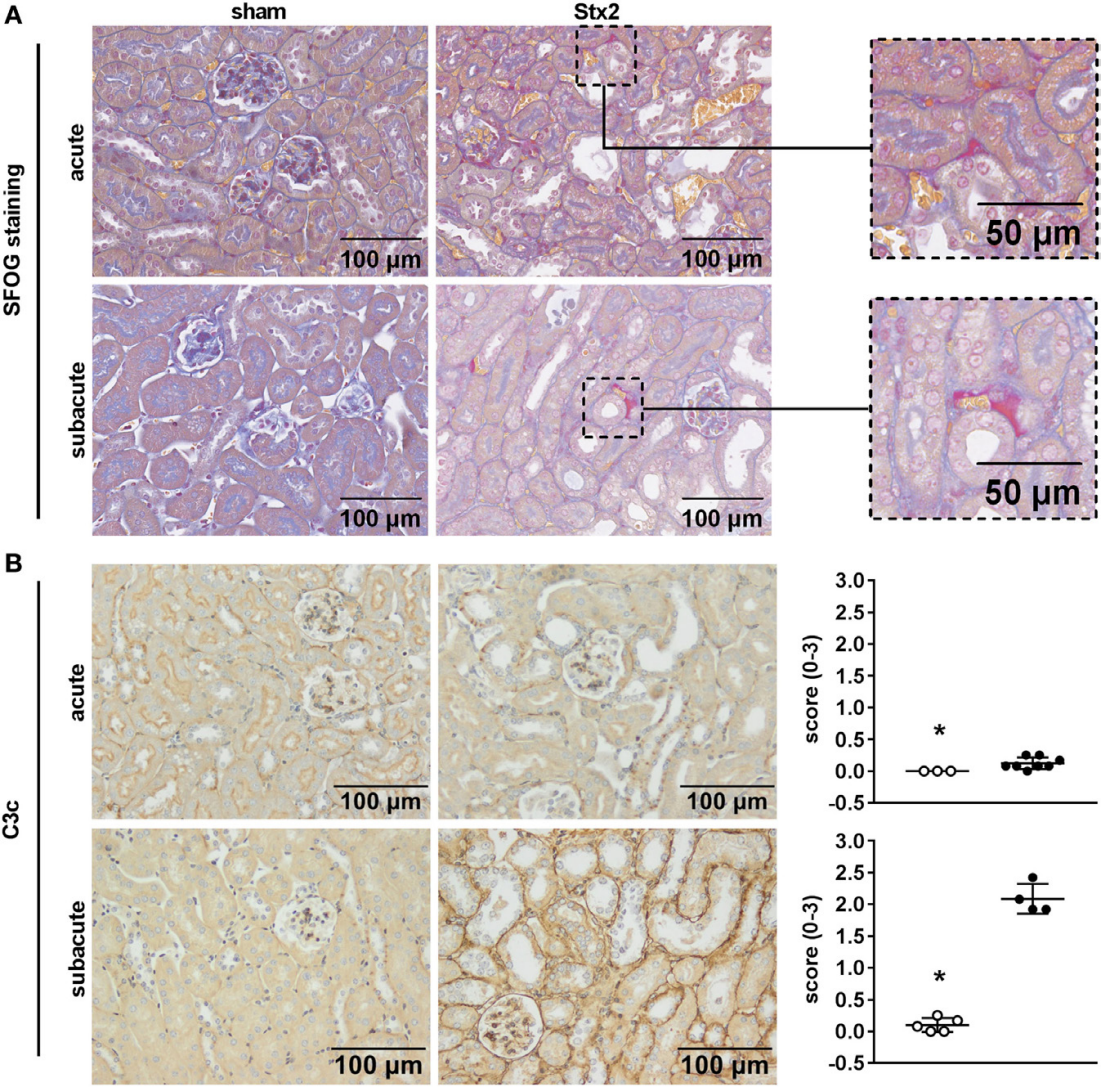
Indicators of thrombus formation and complement activation in C57BL/6J mice subjected to different Stx2 regimens. **(A)** Representative images (scale bar 100 µm) of SFOG staining in renal sections. Thrombus formation was observed in Stx2 groups of both models (see zoomed areas on the right, scale bar 50 µm). **(B)** Representative images (scale bar 100 µm) of immunohistochemical detection and quantification of C3 and C3b complement deposition by C3c staining in renal sections of C57BL/6J wild-type mice are depicted (acute: sham *n* = 3, Stx2 *n* = 8; subacute: sham *n* = 5, Stx2 *n* = 4). *p < 0.05 for sham (white dots) vs. Stx2 (black dots; Mann–Whitney *U*-test).

One hallmark of renal HUS pathology is the formation of microthrombi. Fibrin depositions (bright red/pink) were detected by SFOG staining and found in Stx2-challenged mice of both the acute and the subacute model but not in sham-treated mice (Figure 8A). To investigate a contribution of the complement system in HUS progression, renal sections were stained with an antibody against C3c that not only detects the soluble cleavage product C3c but also C3 and C3b. Compared with sham controls, Stx2-challenged mice showed a significant (acute *p* = 0.0303, subacute *p* = 0.0159) peritubular complement deposition, which was much higher in the subacute model (Figure 8B).

### Effect of Different Stx2 Regimens on Ultrastructural Changes

**Figure 9:**
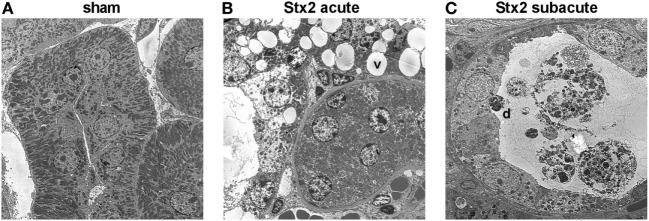
Electron microscopic analysis of kidney tissue from C57BL/6J mice subjected to different Stx2 regimens. Representative ultrastructural images of renal tubules show **(A)** no pathological changes in sham animals, **(B)** prominent vacuoles (v) in the acute and **(C)** pronounced tubular atrophy (d = detached tubular cell) in the subacute model. Magnification: 2,000×; ultrathin sections, *n* = 2 animals were studied per group.

Electron microscopy of kidneys from mice that received Stx2 demonstrated tubular damage in both models. Ultrastructural images from Stx2-challenged mice of the acute model showed extensive vacuolization and less mitochondria compared with sham-treated mice (Figure 9B). Kidneys from Stx2-challenged mice of the subacute model show smaller vacuoles, loss of the brush border, epithelial cell flattening, and more detached necrotic cells indicating higher tubular injury (Figure 9C). No pathological findings were observed in kidneys of sham mice (Figure 9A). Changes of total kidney volume, the total glomeruli count, and tuft volume in the subacute model were further assessed by light sheet fluorescence microscopy (LSFM; Section “Supplementary Results” and Figure S4 in Supplementary Material).

### Effect of Different Stx2 Regimens on Renal Gene Expression

**Figure 10:**
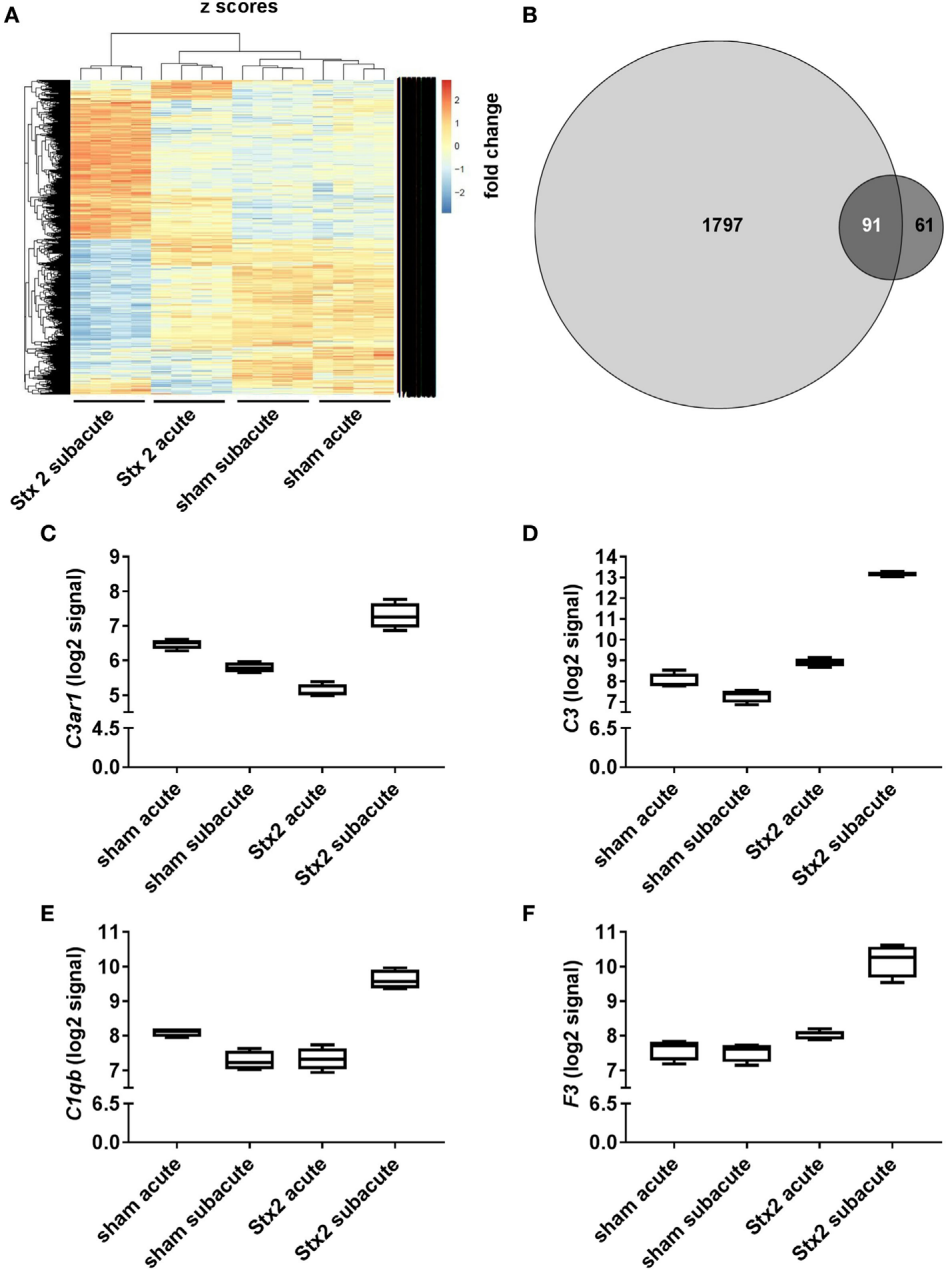
Changes in renal gene expression in response to different Stx2 regimens. **(A)** Heat map of features obtained from ANOVA filtering adjusted *p* < 0.05 comprising *z*-score scaled values. In each group, *n* = 4 animals were studied. **(B)** Proportional Venn diagram of differentially expressed genes according to the limit fold change for acute (dark gray) vs. subacute model (light gray). Number of overlapping genes is highlighted in white. Gene expression data were filtered for candidate genes from complement pathway. Log2 signals for **(C)** complement C3a receptor 1 (*C3ar1*), **(D)** complement factor C3 (*C3*), **(E)** complement C1q B chain (*C1qb*), and **(F)** tissue factor (*F3*) are illustrated by box plots.

A heatmap based on the ANOVA-results of gene expression data and comprising the *z*-scores of the most significant features of the array revealed that all four groups of the experiment are clearly distinguishable by their expression patterns. Hierarchical clustering demonstrated that both sham groups are highly related and the Stx2 acute group also clusters closely with them. The Stx2 subacute group was most distinct (Figure 10A). This was also reflected in the proportional Venn diagram of DEGs for the acute and subacute model (Figure 10B). 152 genes in total were altered in the acute model and 1,888 genes in the subacute model. Of that, 91 genes were altered both in the acute and subacute model. These overlapping DEGs were assigned to different biological processes and molecular functions according to their functional annotation *via* DAVID 6.8 as depicted in Table 1. Both Stx2 regimens led to upregulation of genes associated with immune response, differentiation, proliferation, apoptosis, coagulation, blood pressure, responses to several types of stress, and DNA binding in the whole kidney. Several genes from different metabolic pathways were altered, however, upregulation and downregulation is balanced. Interestingly, a high number of genes for proteins with transporter activity required for normal kidney function and water homeostasis was downregulated in both models of murine HUS. Of note, almost all 91 DEGs common to both models were regulated in the same manner. The only exceptions were *C3ar1* (encoding complement C3a receptor 1) that was downregulated in the acute but upregulated in the subacute model and Gm15889 (predicted gene and protein coding) that was upregulated in the acute and downregulated in the subacute model. Gene expression data were analyzed for further candidate genes from the complement pathway. Apart from *C3ar1, C3* (encoding complement factor C3), *C1qb* (encoding complement C1q B chain), and *F3* (encoding Tissue Factor) were also significantly upregulated in the subacute, but not in the acute model (Figures 10C–F).

**Table 1:** The 91 overlapping differentially expressed genes of the acute and subacute model were assigned to biological process or molecular function and itemized to upregulation and downregulation for each model.

Biological process/molecular function	Stx2 acute		Stx2 subacute	
	Up	Down	Up	Down
**Biological process**				
Angiogenesis	2	2	2	2
Apoptosis	9	2	9	2
Blood pressure	2	–	2	–
Circadian rhythm	1	–	1	–
Coagulation	3	–	3	–
Cytokine signaling	5	2	5	2
Development		2		2
Differentiation	8	2	8	2
Immune response	9	1	9	1
– Of that: chemotaxis	5	1	6	
Inflammatory response	11	3	12	2
Keratinization	1	–	1	–
Metabolism	6	11	6	11
– Of that: cholesterol metabolism	1	2	1	2
– Of that: lipid metabolism	3	4	3	4
– Of that: metabolism of vitamin D	1	2	1	2
– Of that: urea cycle	1	–	1	–
– Of that: steroid metabolism	–	3	–	3
Proliferation	8	1	8	1
– Of that: cell cycle regulation	6	1	6	1
Regulation of pH	1	1	1	1
Response to DNA damage	1	–	1	–
Response to hypoxia	1	4	1	4
Response to molecules of bacterial origin	2	–	2	–
Response to oxidative stress	3	1	3	1
Response to stress	3	–	3	–
**Molecular function**				
Coding unknown proteins	2	1	1	2
Complement activity	–	1	1	–
DNA binding	15	1	15	1
Glycosylation	–	1	–	1
Ion binding	–	1	–	1
Integral membrane component	–	1	–	1
ncRNA	4	1	4	1
Transporter activity	–	17		17
– Of that: ion transport	–	10	–	10
– Of that: urea transport	–	1	–	1
– Of that: toxin transport	–	1	–	1
– Of that: water transport	–	3	–	3
tRNA processing	1	–	1	–

*Almost all genes were regulated in the same manner in both models, exceptions are highlighted by red boxes*.

Functional annotation clustering of the 91 DEGs was performed *via* DAVID 6.8 to identify the most overrepresented biological terms and thereby clarify the biological processes altered by Stx2. 26 annotation clusters were identified. Of these, 12 annotation clusters were significantly enriched (enrichment score ≥ 1.3). These clusters were mainly associated with inflammatory and immune response (4 of 12), transporter activities (3 of 12), and cytokine activity (3 of 12). The remaining two clusters were associated with apoptosis and positive regulation of transcription (Table 2).

**Table 2:** The 12 significantly enriched annotation clusters identified with DAVID 6.8 in the 91 differentially expressed genes of the acute and subacute model.

Annotation cluster	Key words	Enrichment score	Number of genes
1	Inflammatory response (*p* = 0.00003), cytokine activity (*p* = 0.0012), immune response (*p* = 0.028)	3.5	22

2	Apoptosis (*p* = 0.0096)	2.96	11

3	Basolateral plasma membrane (*p* = 0.00097), major intrinsic protein (*p* = 0.038), water channel activity (*p* = 0.048)	2.87	10

4	Cellular response to interferon-gamma (*p* = 0.028), interleukin-1 (*p* = 0.034), or tumor necrosis factor (*p* = 0.77)	2.69	6

5	Cytokine (*p* = 0.0012), growth factor (*p* = 0.37)	2.35	8

6	Cytokine activity (*p* = 0.0012), cytokine (*p* = 0.0012), secreted (*p* = 0 0.017)	2.34	26

7	Response to hypoxia (*p* = 0.075), regulation of blood pressure (*p* = 0.093)	2.16	7

8	Integral component of plasma membrane (*p* = 0.00019), ion transport (*p* = 0.0029), glycoprotein (*p* = 0.014)	1.94	45

9	Domain: leucine zipper (*p* = 0.000074), DNA-binding region: basic motif (*p* = 0.00044), transcriptional factor activity (*p* = 0.049)	1.8	28

10	Epstein–Barr virus infection (*p* = 0.053), NFκB signaling pathway (*p* = 0.091)	1.49	23

11	Response to endoplasmic reticulum stress (*p* = 0.56)	1.34	4

12	Sodium transport (*p* = 0.37)	1.32	3

*Clusters were arranged according to the enrichment score. The most important key words (Benjamini-adjusted *p*-values in brackets) and number of genes in each cluster are given for each cluster*.

The microarray dataset was published in Gene Expression Online and is available under “GSE99229—Alterations in gene expression in response to different regimes of Shiga toxin 2.”

## Discussion

### Reproducible Induction of HUS-Like Disease by i.v. Administration of Stx2

Several groups have attempted, more or less successful, to generate murine models of HUS. Mice were challenged either by gavage of viable STEC [reviewed by Mohawk and O’Brien (34)], by i.p. co-application of a single high dose of lipopolysaccharide (LPS) and Stx2 (31, 32), by i.p. application of a single high dose of Stx2 alone (32), or by multiple i.p. applications of sub-lethal doses of Stx2 (33, 39, 40). In most studies, injection of Stx alone appeared to be insufficient to induce HUS-like disease in mice (31, 32, 41). However, in 2008, Sauter et al. introduced a model where repeated i.p. injections of sub-lethal doses of Stx2 led to the manifestation of HUS-typical symptoms in C57BL/6J mice (33). In our study, acute or subacute progression of HUS-like disease in mice was induced by application of different doses of Stx2, purified from the well-characterized EHEC O157:H7 patient isolate 86-24 originating from a HUS outbreak in the USA (35). Apart from being the causative agent for HUS in humans, this strain reproducibly induced a HUS-like disease in gnotobiotic piglets (22, 24, 25). In contrast to other studies, we injected Stx2 i.v. aiming for rapid delivery and highest bioavailability (42). We used mice aged 10–16 weeks as they resemble human adolescents or young adults (43) which are an important patient subgroup based on the estimation of global STEC-HUS incidence performed by Majowicz et al. (44).

### LPS Does Not Appear to Be a Prerequisite for the Development of HUS in Humans and Mice

The role of LPS in infection-associated HUS is not entirely clear. LPS has been reported to augment Stx-mediated cytotoxicity by cytokine release in mice (45, 46), but does not appear to be prerequisite for HUS development in humans. In STEC-HUS, high serum levels of anti-LPS antibodies (47) and elevated LPS binding protein levels (48) were detectable, but most notably, endotoxemia has not yet been reported. Only in very rare cases of non-STEC-HUS, e.g., caused by infections with *Shigella ssp*. (49), LPS was detectable in the plasma of patients by *Limulus* assay. It was reported previously that Stx2 alone could induce pro-inflammatory transcription events that might contribute to HUS pathogenesis (50, 51). In this study, we avoided the use of additional pro-inflammatory stimuli and focused on pathomechanisms exclusively mediated by Stx2.

### Standardized Surveillance of Stx2-Challenged Mice by a Scoring System Is Necessary

Especially in the early stage, we noticed that disease aggravation occurs with unapparent clinical signs and loss of weight appears as the first symptom. We observed a profound weight loss over time in both models comparable to what has been reported by other groups (32, 33). Interestingly, in the subacute model mice frequently developed a pronounced hind limb clasping reflex in addition to neurological symptoms like tremors and ataxia that have already been described by Sauter et al. (33). To monitor disease progression, we developed and introduced—for the first time—a scoring system based on the activity of mice. It detects critically ill mice that need to be euthanized and, thus, became very important not only for accurate experimentation but also in terms of animal welfare.

### Volume Resuscitation Is a Crucial Measure to Attenuate Hemoconcentration in Mice

As a consequence of the profound hemoconcentration and weight loss observed in pilot experiments, we, for the first time, established a volume resuscitation regimen to prevent severe hypovolemia. While characterizing the acute model, we realized that s.c. administration of 0.5 ml balanced crystalloids twice daily was not sufficient to alleviate hemoconcentration. Therefore, we expanded volume resuscitation in the subacute model to three doses of 0.8 ml daily. Upon hospital admission, STEC-HUS patients frequently show slightly elevated levels of hemoglobin or hematocrit as a consequence of diarrhea-induced dehydration (52, 53). Thus, we assume prerenal pathomechanisms contribute to HUS pathology and might aggravate disease progression. This hypothesis is supported by studies that identified hemoconcentration as an independent risk factor for increased mortality (54) and severity of HUS (53). However, in our study, we aimed to prevent, by volume replacement therapy, the prerenal mechanisms from outweighing the Stx2-related mechanisms. Even though profound hemoconcentration was observed previously in murine HUS models (32), volume resuscitation strategies have thus far not been included in experimental designs.

### The Development of a Photometrical Assay Allows a More Accurate Quantification of Hemolysis

Hemolysis is a defining feature of HUS. Recently, the central role of extracellular “free” heme as a perpetuating factor in life-threatening infections, even with only a very moderate degree of hemolysis, has been acknowledged (55). Here, we determined the degree of hemolysis not only by measuring indirect markers, such as plasma bilirubin and LDH, but also by developing a photometrical assay to quantify free plasma hemoglobin. We detected hemolysis in both models, however, it was more pronounced in the subacute model. Our results are in line with other studies, which observed hemolysis in murine HUS either by simple visual inspection (32) or photometrically (33).

### Persistent Neutrophilia Might Contribute to Disease Progression

In accordance with our recent observations in EHEC-infected gnotobiotic piglets (25) and with the finding by Sauter et al. in Stx2-challenged mice (33), we found low leukocyte counts in both models that were accompanied by persistent neutrophilia. There is some evidence that neutrophilia directly contributes to Stx2 toxicity as mortality and kidney damage are reduced in a murine model of HUS using polymorphonuclear-cell-(PMN)-depleted mice (56). Neutrophil extracellular traps were shown to aggravate intravascular coagulation during sepsis in mice (57) and their degradation appears to be impaired in HUS patients (58), which further underlines the role of neutrophilia in HUS pathogenesis. Furthermore, we demonstrated macrophages and T lymphocyte invasion in the kidneys of mice that underwent the subacute, but not the acute protocol, indicating an activation of the innate and adaptive immune response. The role of mitochondria in immune response was intensively studied during the past years [as reviewed in Ref. (59)]. Distinct changes in the metabolic profile of human endothelial cells in response to Stx2 were shown *in vitro* (60) but not yet assessed *in vivo*. In this context, further analysis on the interrelation between immune response and metabolism might elucidate novel aspects of HUS progression.

### Stx2 Challenge Induced Kidney Dysfunction, Renal Endothelial Damage, and Thrombotic Microangiopathy as Key Features of HUS

Consistent with the literature (31–33), we observed kidney dysfunction following Stx2 challenge, indicated by significant rises of plasma urea and creatinine. Furthermore, we detected—for the first time—significantly higher levels of plasma NGAL in the acute and subacute model, which have also been shown in HUS patients with renal dysfunction (61), but to our knowledge have not been examined in murine models of HUS. Occurrence of renal endothelial damage is crucial for the development of HUS (62, 63). So far, evidence for endothelial damage in mouse models of HUS was mainly provided on an ultrastructural level (31–33). Here, we additionally demonstrated by immunohistochemical assessment and quantification a significant decrease of CD31-positive endothelial cells in both disease models, indicating severe endothelial cell injury. Thrombotic microangiopathy in the kidneys is a hallmark of HUS that was so far only detectable in murine HUS models induced by co-application of Stx2 and LPS (31, 32). It could not be demonstrated in the repetitive Stx2 injection model presented by Sauter et al. (33). However, we were able to demonstrate microthrombi formation in our acute and subacute model.

### In Contrast to Human HUS Tubular Rather Than Glomerular Lesions Predominate in Stx2-Induced Murine HUS

Tubular (64) and glomerular injury (65, 66) contribute to HUS pathology in patients. In rodent models of HUS, tubular rather than glomerular injury is a common finding (41, 67–69) possibly owing to the higher expression of the Gb3 receptor on glomerular cells in humans as opposed to on tubular cells in rodents (70). Nevertheless, some studies found glomerular injury in an ultrastructural analysis (31–33). In both models, we could not detect glomerular injury on the ultrastructural level and we found no significant changes in the kidney volume and total count of glomeruli of mice by LSFM (only assessed in the subacute model). In many former preclinical HUS studies, tubular injury in mice could either not be detected [Stx2/LPS co-administration (70)] or has not been examined [Stx2/LPS co-administration (31, 32), Stx2 model (33)]. However, we observed severe tubular injury in both Stx2 models. The results of ultrastructural and immunohistochemical analyses indicate that tubular damage is more pronounced in the subacute model.

### Apoptosis and Proliferation Only Occur in the Subacute Course of HUS-Like Disease in Mice

We detected elevated apoptosis rates of tubular epithelial cells indicated by significant increase in cleaved caspase 3 staining in the subacute, but not in the acute model. Apoptosis was shown to be profound in tubular epithelial cells in human STEC-HUS, independent from the grade of thrombotic microangiopathy, indicating that it separately contributes to the pathogenesis of tubular injury observed in these patients (64). Although potentially involved in HUS pathogenesis, apoptosis was not yet examined in most murine models of HUS (31–33). Most notably, we demonstrate for the first time that proliferation of tubular epithelial cells is increased in response to Stx2 as indicated by a significantly higher expression of Ki67 in the subacute model. We hypothesize that this effect might be a reactive mechanism to the re-occurring harmful stimuli of multiple Stx2 injections.

### Complement Activation Plays a Major Role in HUS Pathogenesis

Complement activation is a hallmark of HUS pathogenesis in STEC-HUS (71, 72) as well as in atypical HUS (73). Complement activation was found in mouse models of Stx2/LPS co-injection (74) and EHEC infection (75). Here, we demonstrate that a single high dose of Stx2 is not sufficient to provoke complement activation in mice, whereas multiple doses of Stx2 induce profound activation of the complement system as indicated by significant renal C3c deposition. The differences in complement activation in the acute and subacute course of disease were further supported by gene expression data of kidneys.

### Further Characterization of Regulated Pathways Can Potentially Lead to New Insights Into Pathophysiological Mechanisms of HUS

Our observations that 1,888 genes were differentially regulated in the subacute disease model and only 152 in the acute one support our hypothesis of different underlying pathomechanisms. However, differential gene expression analysis of kidneys revealed 91 overlapping genes in significantly enriched categories (e.g., cytokine signaling, apoptosis, immune, and inflammatory response, transporter activity). While changes in renal gene expression following Stx2 or Stx2/LPS co-challenge have already been characterized by Keepers et al. (32), we are the first to demonstrate that also three repetitive sub-lethal doses of Stx2 lead to considerable changes in transcriptional events in a murine model of HUS. The gene clusters we found to be differentially regulated in both models also match the ones proposed by Keepers et al. in a model of LPS/Stx2-co-challenge (32). As we isolated total kidney mRNA, it remains unclear if these changes might be cell-type specific. Furthermore, the more prominent changes in the subacute model might be partially explained by the high amount of invading immune cells that might contribute to changed expression patterns.

## Conclusion

We have established two murine models that allow studying the action of Stx2 in an acute and subacute phase of HUS-like disease. Acute kidney dysfunction and injury, hemolysis, renal endothelial cell damage and microthrombi, as well as neurological symptoms were reproducibly observed in both models as pathognomonic signs of HUS in humans. Most notably, intrarenal changes, such as complement activation, accumulation of macrophages and T lymphocytes, as well as apoptosis and proliferation could only be observed in the subacute model, whereas hypovolemia, as a prerenal pathomechanism, appears to play a major role for the development of kidney dysfunction in the acute model. With this study, we provide murine models of HUS that will serve as suitable and valuable tools to further characterize the pathophysiology of HUS and perform pharmacological trials in HUS and related conditions associated with impaired microcirculation in the kidney.

## Ethics Statement

This study was carried out in accordance with the German legislation and approved guidelines (“Tierschutzgesetz” and “Tierschutz-Versuchstierverordnung”). The animal protocols were approved by the regional animal welfare committee and the Thuringian State Office for Consumer Protection and Food Safety, Bad Langensalza, Germany (registration number 02-058/14).

## Author Contributions

Conception and design of the study: SC; conception and performance of animal experiments: SD, BW, WP, and SC; sample analysis and statistical analysis: SD, WP, BW, CD, MK, and SC; contribution of important intellectual content to histologically analysis: CD and KA; purification and supply of Stx2: FG and WR; LSFM: WP and MG; automated LSFM image analysis: AM and MF; microarray analysis: SD, SL, and SC; drafting the manuscript for important intellectual content: SC, SD, WP, FG, and CD; revising the manuscript prior to submission: SC, SD, CD, FG, PZ, WP, BW, SL, MF, and MG; all authors carefully reviewed and approved the manuscript.

## Conflict of Interest Statement

The authors declare that the research was conducted in the absence of any commercial or financial relationships that could be construed as a potential conflict of interest.
