# Supplementary material for: Modeling Hemolytic-Uremic Syndrome: In-Depth Characterization of Distinct Murine Models Reflecting Different Features of Human Disease
Source: Front Immunol. 2018 Jun 25;9:1459. doi: 10.3389/fimmu.2018.01459 (PMC6026657; doi:10.3389/fimmu.2018.01459)
Supplement: Supplementary file 2 [file Data_Sheet_1.PDF]

Supplementary Material

Modeling hemolytic-uremic syndrome: In-depth characterization of distinct murine models reflecting different features of human disease

Sophie Dennhardt[1,2,3,§]; Wiebke Pirschel[1,2,§]; Bianka Wissuwa[1,2]; Christoph Daniel[4]; Florian Gunzer[5]; Sandro Lindig[1]; Anna Medyukhina[6]; Michael Kiehntopf[7]; Wolfram W. Rudolph[5]; Peter F. Zipfel[8]; Matthias Gunzer[9]; Marc Thilo Figge[3,6,10]; Kerstin Amann[4]; Sina M. Coldewey[1,2,3,*]

[1]Department of Anesthesiology and Intensive Care Medicine, Jena University Hospital, Jena, Germany; [2]Septomics Research Center, Jena University Hospital, Jena, Germany; [3]Center for Sepsis Control and Care, Jena University Hospital, Jena, Germany; [4]Department of Nephropathology, Friedrich-Alexander University (FAU) Erlangen-Nürnberg, Erlangen, Germany; [5]Institute of Medical Microbiology and Hygiene/Institute of Virology, TU Dresden, Dresden, Germany; [6]Applied Systems Biology, Leibniz Institute for Natural Product Research and Infection Biology, Hans Knöll Institute, Leibniz-Association, Jena, Germany; [7]Department of Clinical Chemistry & Laboratory Medicine, Jena University Hospital, Jena, Germany; [8]Department of Infection Biology, Leibniz Institute for Natural Product Research and Infection Biology, Jena, Germany; [9]Institute for Experimental Immunology and Imaging, University Hospital, University Duisburg-Essen, Essen, Germany; [10]Friedrich Schiller University (FSU) Jena, Jena, Germany

[§]S.D. and W.P. contributed equally

*** Correspondence:**
Dr. med. Sina M. Coldewey, PhD,
Department of Anesthesiology and Intensive Care Medicine
Jena University Hospital,
Am Klinikum 1
07747 Jena, Germany
sina.coldewey@med.uni-jena.de

1 Supplementary Methods

1.1 Refinement of scoring system for murine HUS models

The modified score system based on our activity score was partially adapted from the clinical severity score introduced for septic animals (Gonnert et al., 2011) and modified to reflect symptoms occurring in mice that suffer from HUS-like disease (Suppl. Table 1).

Supplementary Table 1: Modified HUS score. Assessment criteria and corresponding points are depicted. Disease score is calculated from the sum of all points for all criteria.

score	points/ criterion	I. activity	II. reaction	III. posture	IV. general symptoms	V. neuro-logical symptoms	VI. fur
**score 1** no signs of illness ≤ 6 points	1	active, strong	curious, fast movements	normal	none	none	shiny, even
**score 2** low-grade ≤ 10 points	2 points for I, II, VI 1 point for III, IV, V	lower activity, occasional interruptions	reduced attention, appropriate reaction	normal	none	none	blunt, adjacent
**score 3** mid-grade ≤ 18 points	3	markedly reduced	reduced attention, delayed reaction	slightly hunched	loss of 10-20% BW in 48 h	beginning hind limb clasping	blunt, slightly ruffled
**score 4** high-grade ≥ 19 points	4	lethargic, no movements	none	strong hunch	loss of > 20% BW in 48 h	pronounced hind limb clasping	blunt, strong piloerection
**score 5** dead	n/a	n/a	n/a	n/a	n/a	n/a	n/a

1.2 Antibodies for immunohistochemistry

Supplementary Table 2: Primary antibodies used for immunohistochemistry. Supplier, working dilution and diluent as well as batch and catalogue number are indicated.

antibody	supplier	dilution	batch	cat. no.
monoclonal anti-F4-80	Serotec, Kidlington, UK	1:100 in Tris-CSA + 1% BSA	1112	MCAP497
monoclonal rabbit anti-CD3	Zytomed Systems, Berlin, Germany	1:100 in Tris-CSA	K793, Clone SP7	RBK024-05
polyclonal rabbit anti-cleaved caspase 3	DCS, Hamburg, Germany	1:200 in Tris-CSA	G721	CI752C002
monoclonal rabbit anti-Ki67	Neomarkers, Freemont, USA	1:200 in Tris-CSA	9106S 906A	RM-9106-S
anti-KIM-1	R&D Systems, Minneapolis, USA	1:500 in Tris-CSA + 1% BSA	KCA0415031	AF1817
monoclonal rat IgG2a anti-CD31	Dianova, Barcelona, Spain	1:100 in Tris-CSA + 1% BSA	15219/01	DIA-310
polyclonal anti-C3c	Dako, Santa Clara, USA	1:100 in Tris-CSA	55806	A0062

1.3 CD31 light sheet fluorescence microscopy and image analysis

Antibody staining, sample preparation, and light sheet fluorescence microscopy (LSFM) were performed as described previously (Klingberg et al., 2017) with slight modifications. Quantification of kidney volume and glomeruli numbers required segmentation of the regions of kidneys and glomeruli. This was carried out automatically by an image analysis algorithm that was adapted from Klingberg et al., 2017. The algorithm was implemented in the programming language “python” (https://www.python.org/) and is available from the authors upon request.

1.4 Algorithm for automated kidney segmentation from light sheet fluorescence microscopy images

In order to detect the kidney region K, the following sequence of steps was performed for each z-layer I_z:

1. Downsizing of each z-layer I_z by the factor of 10 (spline interpolation, order 3) to reduce the computational load and smooth intensity variations (I'_z)
2. Preprocessing of each z-layer I'_z:
   a. Gaussian filter of $I'_z, \sigma = 30$ µm (G_z)
   b. Normalization of G_z to the 99th percentile of its intensity (N_z)
3. Computing the background seeds for watershed segmentation (B), which is done using the maximum projection of the z-stack:
   a. Computing the maximum projection of the middle 90% of all I'_z z-layers (M)
   b. Normalizing the M between the 20th and the 99th percentiles and a Gaussian filter with $\sigma = 5$ µm (N_M)
   c. Computing the foreground seed (F_M):
      i. Otsu thresholding of N_M (T_M)
      ii. Morphological erosion of T_M with a disk of radius 5 pixels (F_M)
   d. Computing the background seed (B_M)
      i. Thresholding of N_M at 10% of its maximum intensity ($T2_M$)
      ii. Morphological dilation of $T2_M$ with a disk of radius 5 pixels (B_M)
   e. Computing the gradient of N_M using the Sobel filter (E_M)
   f. Watershed segmentation of E_M using F_M as the foreground seed and B_M as the background seed (W_M)
   g. Morphological dilation of W_M with a disk of radius 5 pixels (B)
4. Excluding z-layers N_z with low contrast to prevent noise detection: all z-layers are excluded for which $\max\{N_z\} \leq 5 \cdot median\{N_z\}$
5. Computing the foreground seeds for watershed segmentation (F_z)
   a. Otsu thresholding of N_z (T_z)
   b. Morphological erosion of T_z with a disk of radius 5 pixels (T'_z)
   c. Computing of the convex hull of T'_z (H_z)
   d. Morphological erosion of B with a disk of radius 5 pixels (B')
   e. Binary product of H_z and B' (F_z)
6. Computing the gradient of N_z using the Sobel filter (E_z)
7. Watershed segmentation of each z-layer N_z using F_z as the foreground seed and B as the background seed (W_z)
8. Upsizing W_z by the factor of 10 to restore the original size of the z-layer:
   a. Spline interpolation (order 3) of W_z (W'_z)
   b. Otsu thresholding of W'_z to obtain a binary mask (W''_z)
9. Median filter of the resulting binary stack of W''_z layers along the z-axis: the new binary mask K_z is computed as the median of the masks of the 10 neighboring z-layers

1.5 Algorithm for automated glomeruli segmentation from light sheet fluorescence microscopy images

The following sequence of steps was applied to segment the glomeruli regions:

1. Median filtering (3 pixels) of each z-layer I_z
2. Normalization of I_z to its maximum intensity value (N_z)
3. Morphological opening of N_z with a disk of radius 50 μm to obtain the background image (B_z)
4. Subtraction of the background image from N_z: $N_z' = N_z - B_z$
5. Computing the 75th percentile of N'_z in the kidney region ($K_z > 0$) (p)
6. Thresholding of N'_z at $6 \cdot p$: $N''_z = \begin{cases} 1, & if\ N'_z > 6 \cdot p \\ 0, & otherwise \end{cases}$
7. Morphological opening of N''_z with a disk of radius 7.5 μm (G_z) to remove small objects
8. Labelling of connected regions in the combined 3D binary mask of G_z layers
9. Removing connected regions with a volume smaller than $1.4 \cdot 10^4\ \mu m^3$ (radius 15 μm) or larger than $5.2 \cdot 10^5\ \mu m^3$ (radius 50 μm)

1.6 Image reconstruction

For manual image analysis ImageJ (Image Processing and Analysis in Java, http://imagej.nih.gov/ij/) and IMARIS 8.1.2 (Bitplane, Switzerland) were used. 3D visualization of LSFM data was performed with the IMARIS software (Video 1).

2 Supplementary Results

2.1 Effect of different Stx2 regimens on organ function

Plasma ASAT was measured as a marker for general organ injury. The levels in Stx2-challenged animals were comparable to sham animals (Suppl. Fig. 3A, B). Plasma ALAT was measured as a marker for liver dysfunction. The levels in Stx2-challenged animals were comparable to sham animals indicating no liver dysfunction (Suppl. Fig. 3C, D).

2.2 Effect of subacute Stx2 regimen on kidney volume and glomeruli count

LSFM was recently described by Klingberg et al. as a powerful tool to visualize whole kidneys in toxemia models of acute kidney injury (AKI) (Klingberg et al., 2017). In order to clarify whether the intrarenal pathological changes, such as the loss of renal endothelial cells were linked with changes in kidney size, total glomerular count or tuft volume, LSFM of whole organs labelled with anti-CD31 from sham-treated and Stx2-challenged mice of the subacute model was performed. Based on the image data obtained (Suppl. Fig. 4A, Suppl. video) the total kidney volume as well as the total number of glomeruli per kidney was calculated by automated image analysis. The total kidney volume was not altered in Stx2-challenged mice compared with sham-treated mice (210.1 mm^3 vs. 207.9 mm^3; Suppl. Fig. 4B). However, a slight but not significant decrease in the total glomeruli count was observed in Stx2-challenged mice compared with sham-treated mice (22,167 vs. 24,708; Suppl. Fig. 4C). The distribution of tuft volumes of Stx2-challenged mice relative to the distribution in mice of the sham group was mostly unaltered (Suppl. Fig. 4D). Differences were observed in the three smallest tuft volume categories (up to $1x\ 10^5\ \mu m^3$), although they fail to reach statistical significance (Suppl. Fig. 4D).

3 Supplementary Figures

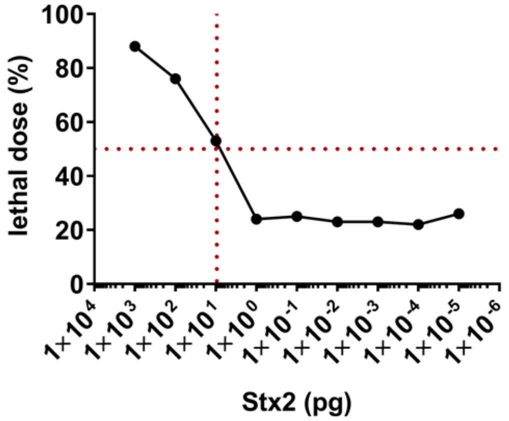

Supplementary Figure 1: Stx2 cytotoxicity in Vero cells. Cytotoxicity was measured by neutral red assay for various Stx2 concentrations and lethal dose was calculated by $OD_{540\text{-}690nm}$. The red lines illustrate the LD_{50}.

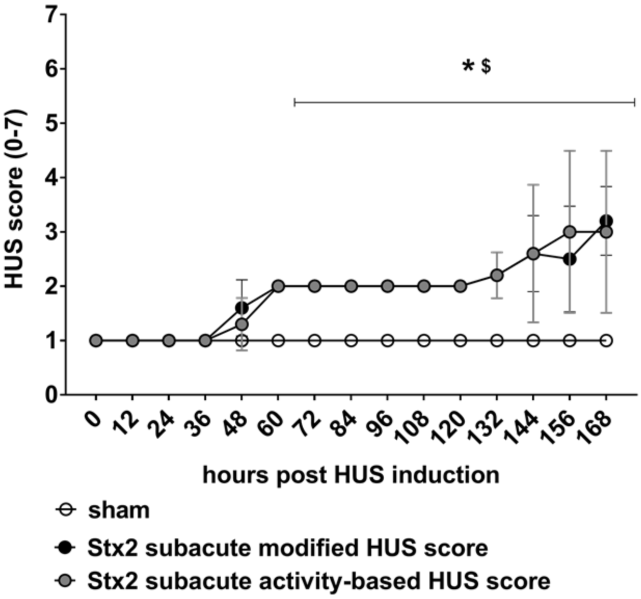

Supplementary Figure 2: Comparison of the activity-based HUS score with the modified HUS score for the subacute model. Clinical presentation of C57BL/6J wild-type mice was assessed by the activity-based score ranging from 1 = very active to 7 = dead and the modified score ranging from 1 = no signs of illness to 5 = dead for the subacute model (sham $n = 8$, Stx2 $n = 10$). Data are expressed as median ± interquartile range for n observations. $^{*}p < 0.05$ for sham vs. Stx2 modified HUS score at each respective time point; $^{\$}p < 0.05$ for sham vs. Stx2 activity-based HUS score at each respective time point (Mann-Whitney-U-test).

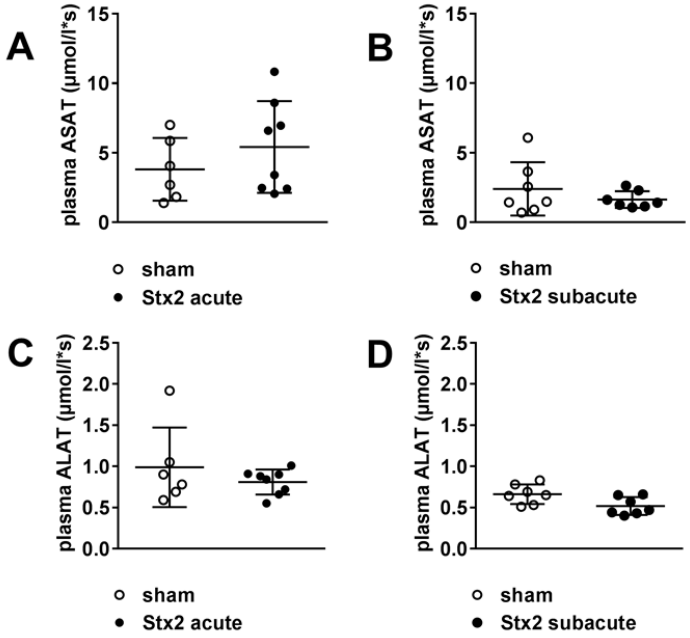

Supplementary Figure 3: Indicators of organ dysfunction in response to different Stx2 regimes. **(A, B)** Plasma ASAT and **(C, D)** plasma ALAT were measured in the **(A, C)** acute (sham $n = 6$, Stx2 $n = 8$) and **(B, D)** subacute model (sham $n = 7$, Stx2 $n = 7$). **(A-D)** Data are expressed as mean ± SD for n numbers of observations. *$p < 0.05$ sham vs. Stx2 (t-test)

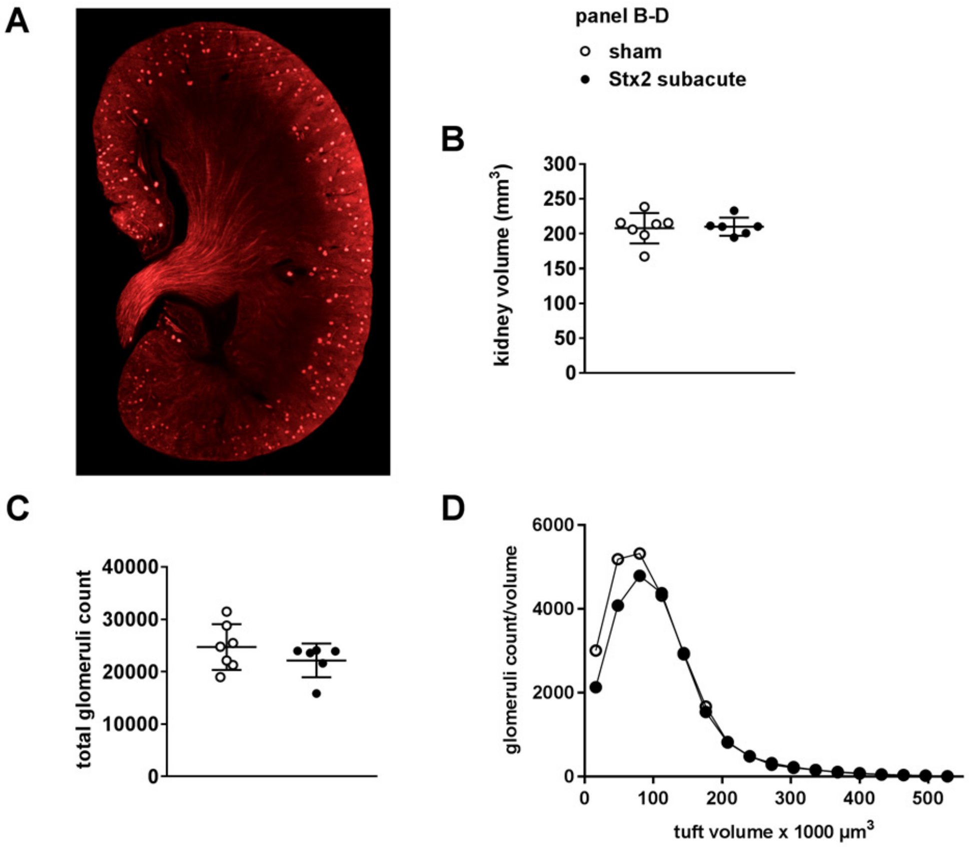

Supplementary Figure 4: CD31 light sheet fluorescence microscopy (LSFM) of kidneys from C57BL/6J sham-treated and Stx2-challenged mice exposed to the subacute HUS model. **(A)** Representative image of LSFM measurements of Stx2-challenged murine kidneys labelled with CD31 (glomeruli are shown as bright red dots) and quantification of **(B)** total volume of two kidneys and **(C)** total number of glomeruli per mouse (two kidneys) by automated image analysis. **(D)** Distribution of tuft volumes of Stx2-challenged mice compared to the distribution in sham mice. Tufts were individually sized by voxel counting. **(B-D)** Data are expressed as mean ± SD (sham $n = 7$, Stx2 $n = 6$).

4 Supplementary video

Video 1: Whole organ imaging by LSFM of a sham kidney. 3D-reconstitution of a cleared healthy kidney. To visualize all glomeruli in the kidney the specific endothelial antibody staining (CD31, red) was highlighted in a 3D-reconstitution of the whole-kidney LSFM *Z*-stack using the Spots tool of Imaris. All determined glomeruli are shown as light grey dots.

5 Supplementary References

Gonnert, F.A., Recknagel, P., Seidel, M., Jbeily, N., Dahlke, K., Bockmeyer, C.L., Winning, J., Lösche, W., Claus, R.A., and Bauer, M. (2011). Characteristics of clinical sepsis reflected in a reliable and reproducible rodent sepsis model. *J Surg Res* 170**,** e123-134.

Klingberg, A., Hasenberg, A., Ludwig-Portugall, I., Medyukhina, A., Mann, L., Brenzel, A., Engel, D.R., Figge, M.T., Kurts, C., and Gunzer, M. (2017). Fully Automated Evaluation of Total Glomerular Number and Capillary Tuft Size in Nephritic Kidneys Using Lightsheet Microscopy. *J Am Soc Nephrol* 28**,** 452-459.
